# Diversity, structure and convergent evolution of the global sponge microbiome

**DOI:** 10.1038/ncomms11870

**Published:** 2016-06-16

**Authors:** Torsten Thomas, Lucas Moitinho-Silva, Miguel Lurgi, Johannes R. Björk, Cole Easson, Carmen Astudillo-García, Julie B. Olson, Patrick M. Erwin, Susanna López-Legentil, Heidi Luter, Andia Chaves-Fonnegra, Rodrigo Costa, Peter J. Schupp, Laura Steindler, Dirk Erpenbeck, Jack Gilbert, Rob Knight, Gail Ackermann, Jose Victor Lopez, Michael W. Taylor, Robert W. Thacker, Jose M. Montoya, Ute Hentschel, Nicole S. Webster

**Affiliations:** 1 School of Biological, Earth and Environmental Sciences, Centre for Marine Bio-Innovation and School of Biotechnology and Biomolecular Sciences, University of New South Wales, Sydney, New South Wales 2052, Australia; 2 The Environment Institute and School of Biological Sciences, University of Adelaide, Adelaide, South Australia 5005, Australia; 3 Ecological Networks and Global Change Group, Experimental and Theoretical Ecology Station, Centre National de la Recherche Scientifique, Moulis 09200, France; 4 Institute of Marine Sciences, CSIC, 08003 Barcelona, Spain; 5 Department of Biology, University of Alabama at Birmingham, Birmingham, Alabama 35487, USA; 6 School of Biological Sciences, University of Auckland, Auckland 1010, New Zealand; 7 Department of Biological Sciences, University of Alabama, Tuscaloosa, Alabama 35487, USA; 8 Department of Biology and Marine Biology, and Center for Marine Science. University of North Carolina Wilmington, 5600 Marvin K. Moss Lane, Wilmington, North Carolina 28409, USA; 9 NAMRA and the Research Institute for the Environment and Livelihoods, Charles Darwin University, Darwin, Northern Territory 0810, Australia; 10 Halmos College of Natural Sciences and Oceanography, Guy Harvey Oceanographic Center, Nova Southeastern University, Dania Beach, Florida 33004, USA; 11 Microbial Ecology and Evolution Research Group, Centre of Marine Sciences, Algarve University, 8005-139 Faro, Portugal; 12 Institute of Chemistry and Biology of the Marine Environment, ICBM, University of Oldenburg, Oldenburg 26111, Germany; 13 Department of Marine Biology, Leon Charney School of Marine Sciences, University of Haifa, Haifa 3498838, Israel; 14 Department of Earth and Environmental Sciences and GeoBio-CenterLMU, Ludwig-Maximilians-Universität, Munich 80539, Germany; 15 Department of Ecology and Evolution, Department of Surgery, University of Chicago, Chicago, Illinois 60637, USA; 16 Argonne National Laboratory, Argonne, Illinois 60439, USA; 17 Departments of Pediatrics and Computer Science and Engineering and Center for Microbiome Innovation, University of California at San Diego, 9500 Gilman Drive, La Jolla, California 92093, USA; 18 Department of Ecology and Evolution, Stony Brook University, Stony Brook, New York 11794, USA; 19 GEOMAR Helmholtz Centre for Ocean Research Kiel, Kiel 24105, Germany; 20 Australian Institute of Marine Science, Townsville, Queensland 4816, Australia

## Abstract

Sponges (phylum Porifera) are early-diverging metazoa renowned for establishing complex microbial symbioses. Here we present a global Porifera microbiome survey, set out to establish the ecological and evolutionary drivers of these host–microbe interactions. We show that sponges are a reservoir of exceptional microbial diversity and major contributors to the total microbial diversity of the world's oceans. Little commonality in species composition or structure is evident across the phylum, although symbiont communities are characterized by specialists and generalists rather than opportunists. Core sponge microbiomes are stable and characterized by generalist symbionts exhibiting amensal and/or commensal interactions. Symbionts that are phylogenetically unique to sponges do not disproportionally contribute to the core microbiome, and host phylogeny impacts complexity rather than composition of the symbiont community. Our findings support a model of independent assembly and evolution in symbiont communities across the entire host phylum, with convergent forces resulting in analogous community organization and interactions.

## Abstract (web summary)

Sponges are early-diverging marine organisms that establish complex symbioses with microorganisms. Here, Thomas *et al.* analyse the microbial communities associated with 81 species of sponges from around the world, shedding light on the ecological and evolutionary drivers of these host-microbe associations.

## Graphical abstract

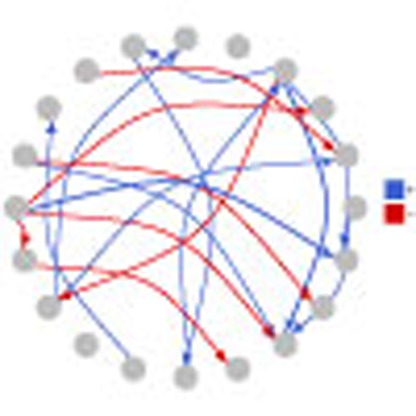

Microbial symbionts are essential for the function and survival of multicellular eukaryotes, ranging from humans to invertebrates to plants^1^,^2^,^3^,^4^. Most symbioses involve complex communities of microorganisms, often comprising a large phylogenetic breadth of microbial diversity associated with a single host organism. Many factors, including host-derived nutrients, chemico-physical characteristics (for example, pH) and host properties (for example, immune response), determine the composition and structure of symbiont communities over time and space. However, the evolutionary and ecological drivers of symbiont composition in animals and plants remain largely unknown^5^.

Sponges are among the most ancient living Metazoa and generally form symbiotic relationships with complex communities of microorganisms^6^,^7^,^8^. Sponges can maintain highly diverse, yet specific symbiont communities, despite the constant influx of seawater microorganisms resulting from their filter-feeding activities^9^. These symbioses are known to be at least partially underpinned by metabolic exchange between symbiont and host, including nitrogen cycling, CO_2_ fixation, secondary metabolite production, and uptake and conversion of dissolved organic matter^10^,^11^,^12^. In this respect, sponge symbionts perform analogous functions to the symbionts found in mammalian guts and plants^5^. Therefore sponge-microbe symbioses represent an ecologically relevant example of host–microbe interactions in an early-diverging metazoan clade.

While the diversity of sponge symbionts has been extensively addressed using molecular tools, comparative work has been hindered due to methodological differences in sampling, sample processing and data analyses^12^,^13^,^14^. Large-scale efforts, such as the Human Microbiome Project^15^ and the Earth Microbiome Project^16^, have standardized these technical aspects to reliably and consistently describe patterns of microbial diversity and composition. These efforts have generated a large knowledge base for host-associated microbiomes of vertebrates, and especially humans, but equivalent data sets for invertebrates are missing. To gain critical insights into the evolution and complexity of symbiotic interactions, we require a greater understanding of the properties and origins of microbial symbioses in early-divergent Metazoa. Furthermore, microbiome research has primarily focused on within-species comparisons, in particular humans, or the comparative analysis of microbiomes of very disparate host organisms (for example, plants versus mammalian guts)^5^. However, to define important aspects for the evolution of microbial symbiosis, a deeper understanding of symbiont communities in closely related host species within defined phylogenetic clades (for example, a single phylum) is required.

Here we provide a comprehensive analysis of microbial symbiont communities associated with 81 species from the phylum Porifera. Through a community effort, a total of 804 sponge samples were collected from the waters of 20 countries bordering the Atlantic, Pacific and Indian Oceans as well as the Mediterranean and Red Seas, primarily from shallow water habitats. For environmental comparison, we simultaneously collected 133 seawater and 36 sediment samples as potential sources or sinks of microorganisms associated with sponges^9^. Microbial community composition for each sample was determined using standardized DNA extraction and 16S rRNA gene-sequencing protocols established by the Earth Microbiome Project^16^. With this extensive data set, we aimed to define the diversity, variability, specificity and similarity of symbiont communities across the phylum Porifera and determine the interaction patterns and evolutionary forces that shape their complexity and composition.

## Results

### Symbiont complexity varies greatly across the Porifera

**Figure 1:**
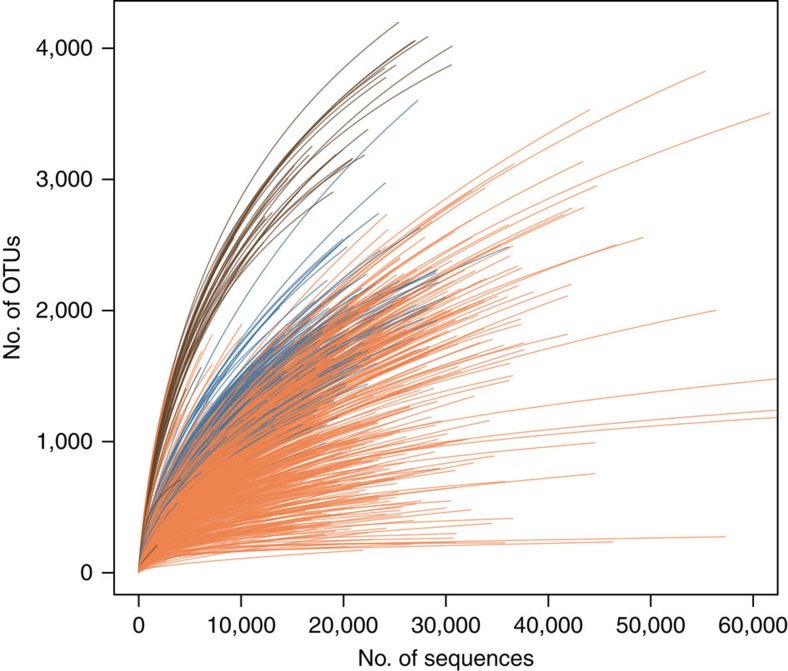
Richness of individual samples from microbial communities in seawater, sediments and sponges. Rarefaction curves of 16S rRNA gene diversity are shown for seawater (blue), sediment (brown) and sponge (orange) samples.

Richness of microbial symbiont communities varies widely across different host species within the phylum Porifera (Fig. 1; Supplementary Data 1). Complexity (as assessed by number of OTUs) ranges from 50 to 3,820 genetically distinct symbionts per host. Seawater operational taxonomic units (OTUs) were removed from sponge samples as they were considered likely to represent ‘environmental contaminants' obtained during filter feeding and sampling (see Methods for details). The large richness estimates are unlikely to be inflated by sequencing errors as approximately one-third of samples reached complete saturation (Fig. 1). Variation of richness across the sponge samples contrasted with the more consistent richness estimates found within seawater and sediment samples (Fig. 1, Supplementary Data 1). The most diverse sponge samples approach the microbial richness found in seawater or sediment, however, most sponge species appear to have somewhat less complex communities than the other two habitats.

**Figure 2:**
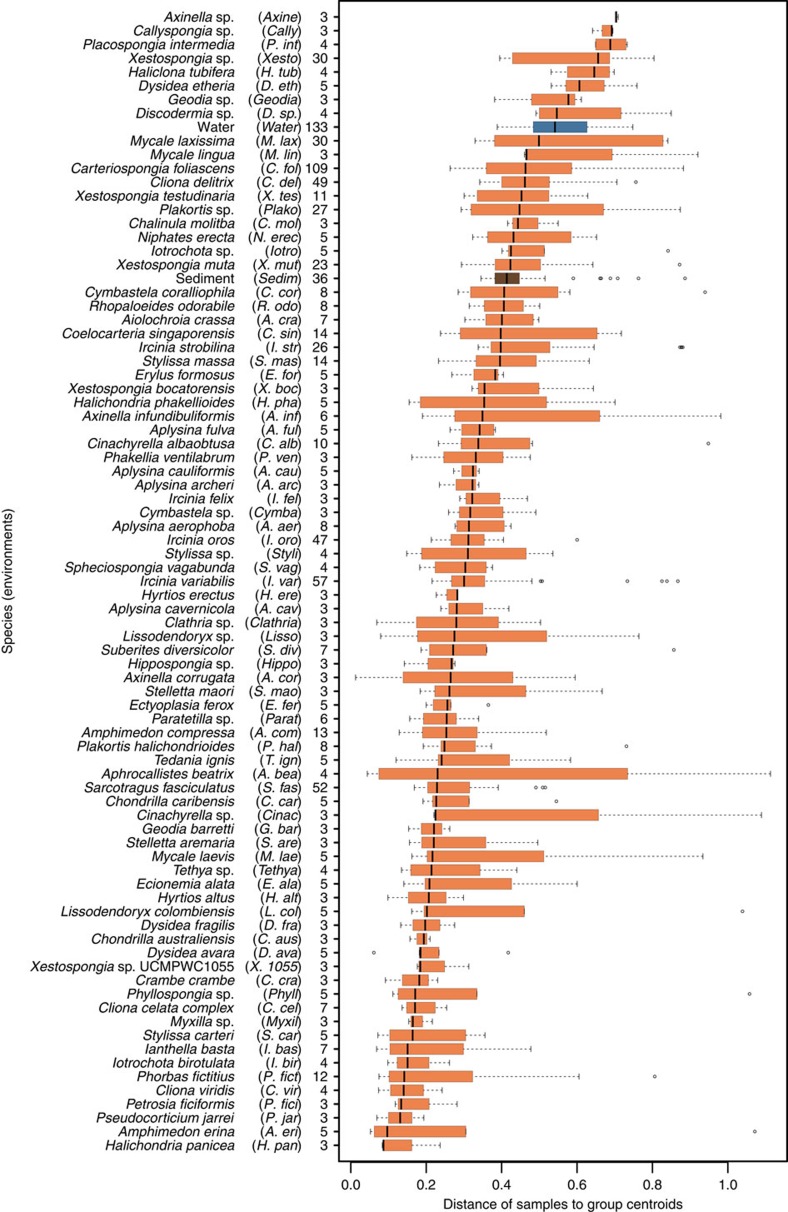
Variability of symbiont communities. Intraspecific community dissimilarity measured as distance of samples to group centroids for 16S rRNA gene composition of different sponge species (orange) and habitats (blue: seawater; brown: sediment). Vertical bar represent the median, the box represent the first to third quartiles and whiskers show the lowest or highest datum within 1.5 times the interquartile range of the lowest and upper quartile, respectively. Names in brackets represent the abbreviations used in all subsequent figures. The number behind the brackets refers to the number of individual samples analysed per sponge taxon, seawater or sediment.

For symbiont communities of the phylum Porifera we observed a continuum of intraspecific dissimilarities across all species investigated (Fig. 2). Variability of symbiont communities between individuals of the same host species is indicative of the nature and strength of host–symbiont interactions, ranging from obligate to facultative^17^,^18^. Thus low variability would indicate that only specific symbionts can interact with the host (high specificity), while a relaxed pressure on the interaction would result in higher variability of symbionts among specimens of the same sponge species. Compared with planktonic communities, most sponges maintain low variability within communities (Fig. 2). Variability was also found to be independent of symbiont diversity or richness (Supplementary Fig. 1). This indicates a generally restrictive or selective habitat or interactions at the host species level, for both diverse and more depauperate symbiont communities.

**Figure 3:**
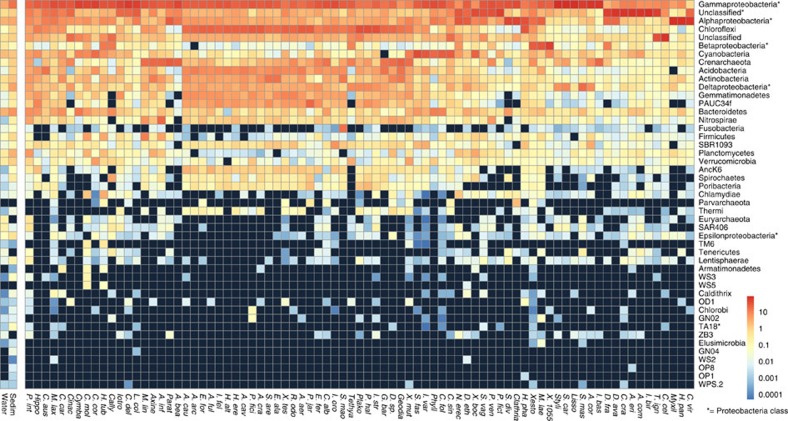
Taxonnomic profile of microbial communities. Average phylum-level taxonomic profile of microbial symbiont communities in 81 different sponge species, seawater and marine sediments. Colour scale shows relative abundance in percentage within each host species. The phylum Proteobacteria is shown as individual classes (including unclassified Proteobacteria), which are indicated by an asterisk. Black squares indicate zero counts. Columns and rows of the heatmap are ordered by Bray–Curtis dissimilarity of their taxonomic profiles (except for seawater and sediments). Sponge species abbrevations are outlined in Fig. 2.

The human microbiome is dominated by four phyla (Firmicutes, Bacteroidetes, Actinobacteria and Proteobacteria) and this phylum-level trend has also been observed in other mammals^19^. In contrast, only the phylum Proteobacteria (especially classes Alpha- and Gammaproteobacteria) was dominant in most sponges species analysed here, with Chloroflexi, Cyanobacteria and Crenarchaeota occasionally reaching high relative abundances (∼10%). Nevertheless, sponges host a high diversity of phyla (albeit at low relative abundances), with over 32 phyla and candidate phyla regularly reported to associate with sponges^20^ and a further 6 phyla and 14 candidate phyla recently reported as part of the rare community using a deep Illumina sequencing approach^21^. In the current study, we detected 41 phyla (including candidate phyla) with all sponges hosting members of at least 13 different phyla (Fig. 3).

### Sponges harbour an exceptional microbial diversity

**Figure 4:**
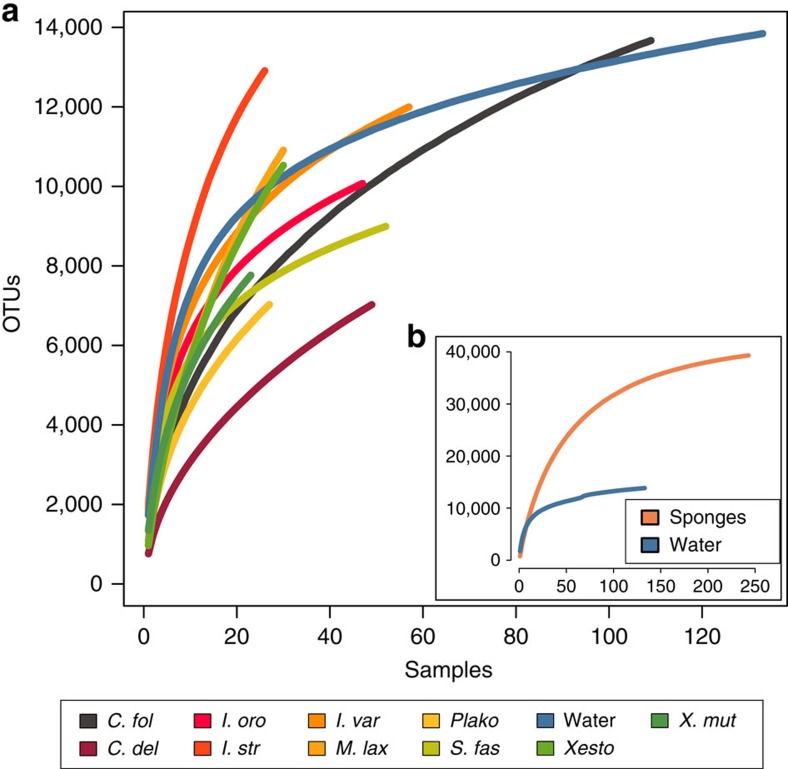
Combined richness of microbial communities in seawater, sediments and sponges. Rarefaction analysis of 16S rRNA gene diversity of microbial communities in sponges and seawater. (**a**) Rarefaction curves for sponge species with more than 20 replicate samples as well as seawater from all sampled geographic regions. OTU diversity is at 97% sequence identity cutoff. (**b**) Rarefaction analysis of all sponge species with three randomly selected samples per sponge species. Sponge species abbrevations are outlined in Fig. 2.

High sample replication (*n*>20) employed in this study facilitated estimation of total microbial richness for specific sponge species and seawater. Analysis of the 133 surface seawater samples (collected here from disparate geographic areas, including Spain, Florida, Puerto Rico, Sweden, Mexico, Bahamas and Australia) showed that the combined planktonic richness in these regions approaches 15,000 OTUs (at 97% sequence identity) (Fig. 4). This estimate lies between the ∼20,000 and ∼9,000 predicted OTUs (at 97% sequence identity) found in surface waters of the coastal and open ocean, respectively, as part of the International Census of Marine Microbes (ICoMM)^22^, which was based on pyrosequencing analysis of the V6 region of the 16S rRNA gene. However, the estimated planktonic richness in this and our study is lower than the 29,457 OTUs (at 97% sequence identity) recently reported using Illumina amplicon sequencing of seawater^21^ or the 37,470 OTUs estimated from metagenomic sequencing of the global Tara Oceans samples^23^, with the higher richness in the latter two studies likely explained by the inclusion of deep-water samples. Remarkably though, richness estimates show that a single sponge species can harbour as many different OTUs as might be expected from the surrounding seawater. For example, *Carteriospongia foliascens* and *Ircinia variabilis* (*n*≥50 individuals across their sampled biogeographic distribution) contain more than 12,000 OTUs (Fig. 4). Similar richness projections were observed for the species *Cliona delitrix*, *Ircinia strobilina*, *Ircinia oros*, *Mycale laxissima*, *Plakortis halichondrioides*, *Sarcotragus fasciculatus*, *Xestospongia* sp. and *Xestospongia muta*, which were each sampled between 20 and 50 times (Fig. 4).

**Figure 5:**
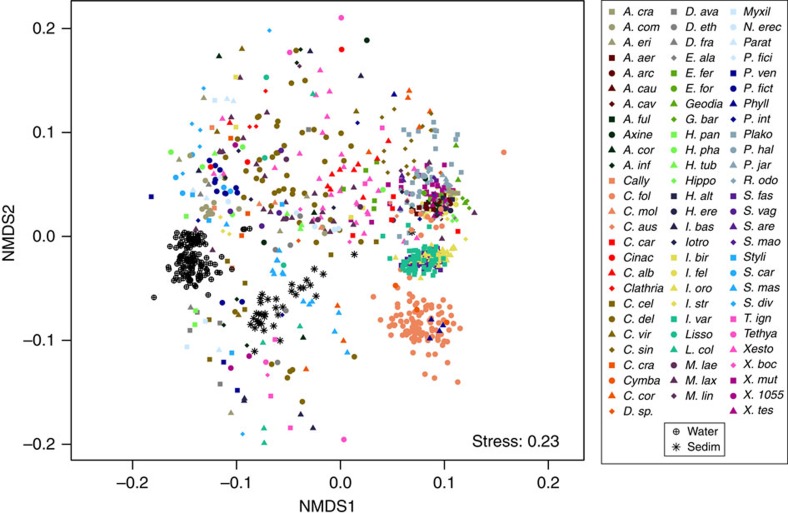
Community similarity for microbial communities in sponges, seawater and sediments. Clustering was performed using multi-dimensional scaling of Bray–Curtis distances. Sponge species abbrevations are outlined in Fig. 2.

Limited overlap in microbiome structure was observed between different sponge species or between sponges and the seawater and sediment samples (Fig. 5). Thus, considering all OTUs discovered across the 804 sponge samples that included 81 different species, richness estimates approach a value of 40,000 OTUs (Fig. 4b). The 81 sponge species analysed here represent only a tiny fraction of the 8,553 described sponge species (and likely a higher number when considering undescribed species)^24^ suggesting that sponge-associated (and likely other host-associated) communities are a significant global source of unique microbial diversity.

### Symbiont communities consist of generalists and specialists

**Figure 6:**
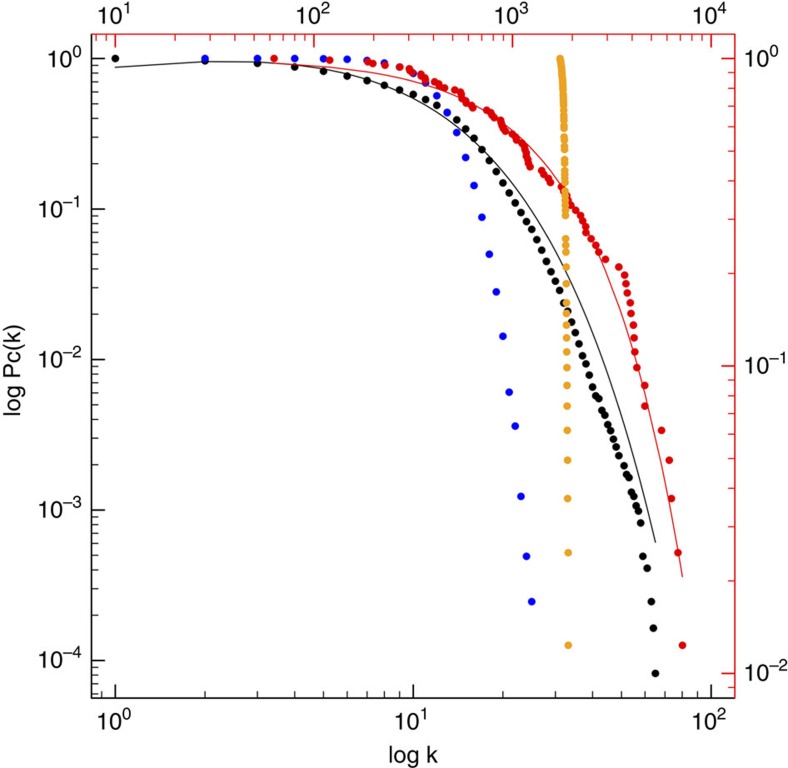
Cumulative degree distributions for OTUs (black dots, bottom and left axes) and sponges (red dots, top and right axes). Black dots correspond to the number of different host species (*k*) that contain a given OTU, represented as the cumulative probability of finding an OTU in the network with *k* or less-associated hosts (Pc(*k*)). Red dots correspond to the number of different OTUs (*k*) found in a given host species, represented as the cumulative probability of finding a sponge host with *k* or less-associated OTUs (Pc(*k*)). The OTU degree distribution followed a truncated power-law Pc(*k*)=*k*^−0.32^ × *e*^−(*k*/7.44)^, while the sponge degree distribution followed an exponential given by Pc(*k*)=*e*^−(*k*/1,849)^. Blue and orange dots correspond to random degree distributions for OTUs and sponges, respectively, where the number of nodes and links from the empirical distribution is kept constant.

To better understand the distribution of symbionts across the Porifera, we constructed a global bipartite network using the associations between OTUs and individual sponge species. The structure of this network differs greatly from what would be expected if connections between sponges and OTUs were randomly assigned (Fig. 6). This suggests that assembly mechanisms (such as ecological and evolutionary processes) are behind the structure of this network of interactions, as has been suggested for other types of networks of ecological interactions^25^.

The cumulative probability of finding an OTU in the network with *k* or less-associated hosts revealed a skewed degree distribution following a truncated power-law with an exponential cutoff at 7.44, almost half the number of host species an OTU is expected to interact with (the average number of hosts a given OTU is found in is 12.13) (Fig. 6). This shows that the majority of symbiont OTUs have a small number of connections and only a few OTUs are very well connected. The majority of OTUs are thus specialists (that is, found in only one or a few sponge species), while only a few are truly cosmopolitan (that is, found across many sponge species). Importantly, the degree distribution for the subset of OTUs belonging to previously defined sponge-specific sequence clusters follows the same distribution as the whole (see below).

The cumulative probability of finding a sponge host with *k* or less-associated OTUs also follows a skewed degree distribution with exponential decay. A large fraction (>50%) of species harbour a symbiont diversity between ∼60 and ∼1,800 distinct taxa, while a small fraction of sponge species can harbour up to ∼7,000 OTUs (see also above). Skewed degree distributions have been identified in several types of ecological networks, and are linked to important properties of ecological communities, such as their robustness to species loss and their stability over time^25^. Our results suggest that ecological communities formed between microbial symbionts and their sponge hosts display similar patterns, which may be linked to their ability to maintain important functions at both the host and ecosystem levels^12^.

**Figure 7:**
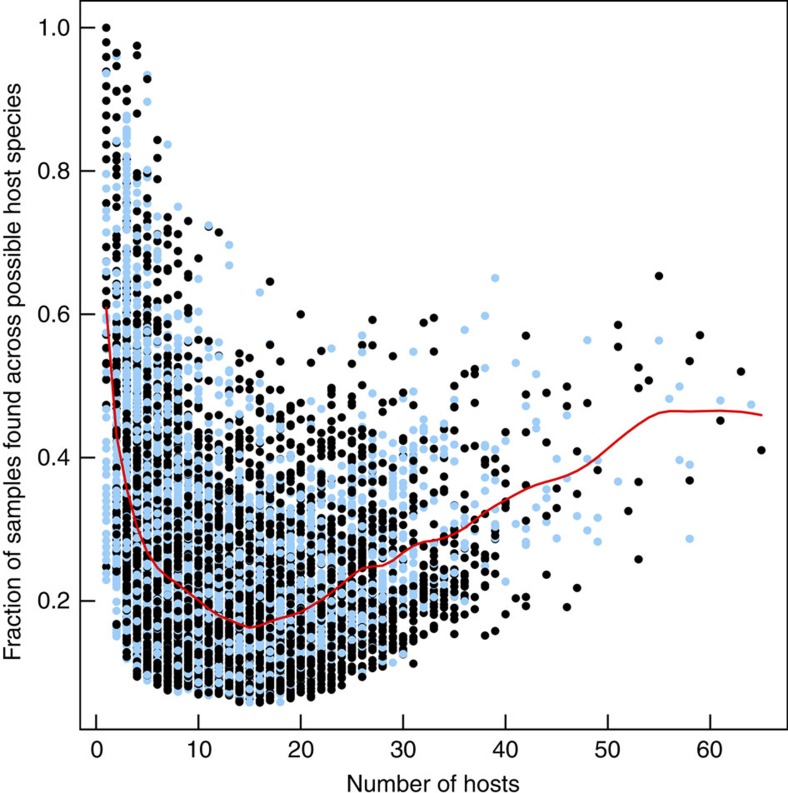
Prevalence of symbionts across different degrees of host-specificity. Number of host species (degree) containing a given bacterial OTU in the bipartite sponge versus symbiont OTU network plotted against the fraction of individual samples where each OTU has been found among all the samples from their known host species. Each point represents an OTU and the red line is a smoothing spline fit to the data (see Methods). Blue dots represent OTUs that belong to sponge-specific sequence clusters (see Results, section ‘Sponge-associated diversity is enriched in specific sequence clusters').

To further investigate the specialization of OTUs in our interaction network, we analysed how consistently they are found across individual replicates of any given host species. Both highly specialized (defined here as those found in less than five different host species) and generalist OTUs (defined here as those found in more than 50 different host species) are present in a large fraction of the biological replicates of their respective host species (Fig. 7; Supplementary Fig. 2). In contrast, a large proportion of OTUs with an intermediate degree of host association (between 5 and 50 host species) can be considered as opportunistic taxa, associated with only a few biological replicates of multiple host species. Thus, symbiont communities within the phylum Porifera are characterized by a combination of highly generalist and truly specialist community members. Our analysis showed that generalists are cosmopolitan not only qualitatively (that is, present in a large number of species), but also quantitatively (that is, consistently present in a large fraction of individuals of those host species). To our knowledge, such patterns have not previously been observed for ecological networks, as it has traditionally been difficult to undertake repeated measures of many individuals across multiple host species.

### Generalist symbionts comprise the core sponge microbiome

**Figure 8:**
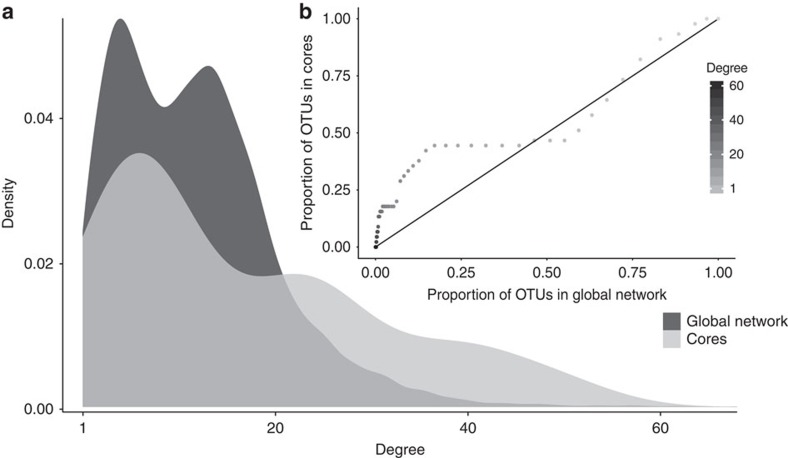
Representation of generalist and cosmopolitan OTUs within the global network and aggregated core. Frequency (density) distribution of degrees for the global bipartite network (dark grey) and the aggregated OTU cores (light grey) (**a**) and the proportion of OTUs with certain degree or higher present in both sets (**b**). In **b**, the *x* axis shows the proportion of OTUs in the global bipartite network, while the *y* axis shows the proportion of OTUs in the aggregated cores. The 1:1 line indicates the expected distribution, if degrees were evenly distributed across the global bipartite network and the aggregated cores. This analysis reveals an over-representation of generalist and cosmopolitan OTUs within the aggregated core, with the break occuring at *k*>12.

Considering the existence of generalist (that is, cosmopolitan) sponge OTUs, we queried their relative contribution to the core microbiome of any individual species. Here we define a core membership as any OTU that is present in at least 85% of the replicates for any single host species. To effectively model population dynamics of these OTUs, we identified host species with a sufficiently large number of replicates (here ≥47) across the entire data set. We identified five host species (*Carteriospongia foliascens, Cliona delitrix, Ircinia oros, Ircinia variabilis* and *Sarcotragus fasciculatus*) that fit this requirement and observed cores ranging in size from 7 to 20 OTUs. The proportion of OTUs with a certain degree (number of connections to different sponge species) or higher, and the frequency distribution of degrees were compared for all OTUs present in the global bipartite network and the aggregated subset of OTUs present in all five core microbiomes (Fig. 8). The core OTUs aggregated from all five sponge species showed an uneven distribution of degree frequencies. Core OTUs are primarily generalist and cosmopolitan (high degree) OTUs, while specialist (low degree) and intermediate degree OTUs are under-represented. This shows that highly connected OTUs in the global bipartite network also tend to comprise a larger fraction of the core for each of the host species investigated here.

### Strong density dependence and weak, unidirectional interactions

Of particular interest is whether these core OTUs and their local interactions are important for the overall dynamics of the symbiont populations within each host species. For instance, density dependence (that is, the growth rate of a population is controlled by its density) has a strong effect on community dynamics, with stabilizing effects on population fluctuations^26^. To disentangle the complex nature of microbe–microbe interactions within our five sponge hosts described above, we applied a statistical framework^27^ that models population dynamics of the Lotka–Volterra type and allows us to decouple the variation in relative abundance of populations into contributions of (i) inter-specific interactions, (ii) density dependence and (iii) environmental stochasticity. Population dynamics are sensitive to both non-modelled environmental stochasticity and modelled fluctuating environmental conditions^27^. However, in this study, the environment is considered as fixed due to replicates being sampled from similar environments during the same time period (see Supplementary Data 2 and also (ref. 28) for temporal stability of symbionts in *Ircinia oros, Ircinia variabilis* and *Sarcotragus fasciculatus*), hence population dynamics are considered to be influenced solely by environmental stochastic processes and species interactions.

Density-dependent processes were found to explain the majority of variation in the relative abundance of core OTUs across biological replicates, followed by stochastic mechanisms (Supplementary Table 1). Only a small proportion of variance (3–8% across hosts) is explained by inter-specific interactions (Supplementary Table 1). It should, however, be noted that the contribution of inter-specific interactions may be larger because we are missing those interactions excluded from the cores (that is, interactions with more opportunistic OTUs).

Although inter-specific interactions contribute little to the dynamics of the core microbiomes, it is still important to investigate the nature and strength of these interactions as, for example, antagonism (that is, competition) and mutualism are known to differ in how they affect population and community stability^29^. Both empirical and theoretical studies in community ecology demonstrate that distributions skewed towards many weak and a few strong interactions enhance both population and community stability, and may arise during the assembly of persistent communities^30^,^31^. Similarly, mutualism or skewed interactions only affecting one interacting partner (that is, amensalism and commensalism) have been shown to promote diversity and lead to community stability^32^,^33^.

**Figure 9:**
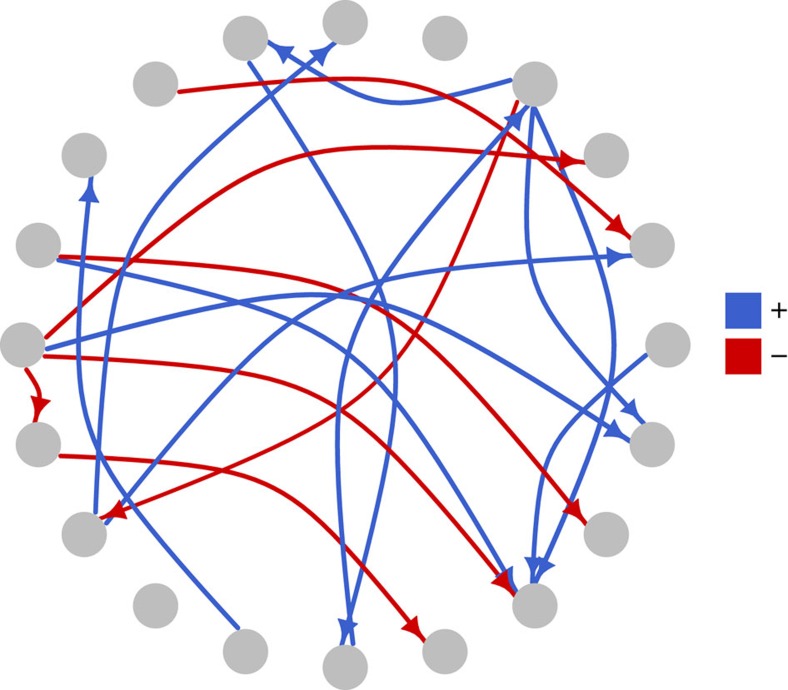
Representative network for the core microbiome of *Ircinia oros*. Each node corresponds to a single OTU, and links illustrate the most probable inter-specific interactions (see Supplementary Fig. 5). Positive and negative interactions are depicted in blue and red, respectively. None of the inter-specific interactions are bidirectional, indicating either amensal (−, 0) or commensal (+, 0) interactions. For full taxonomic information of the nodes refer to Supplementary Fig. 7 and Supplementary Data 3).

A number of indices were calculated for each core microbiome (Supplementary Table 2). Despite some variability in OTU number and linkages across different hosts, connectance (defined as the fraction of realized links among all possible links) was consistently low, ranging between 4.5 and 7.5%. We find that all cores are characterized by very few strong and many weak interactions (Supplementary Fig. 3). Moreover, cores are distingished by a mixture of positive and negative interactions with amensalism and commensalism as a signature rather than competition and/or mutualism (Fig. 9 illustrates this using the example of *Ircinia oros*; see Supplementary Figs 4–7 for further details and other sponge species). Across hosts, we observe that the most probable links are generally negative, although as the core size increases, the fraction of positive inter-specific interactions increases. Interestingly, we find that some OTUs, which are highly connected in the global bacteria-sponge (bipartite) network, are also highly connected within the core network. This suggests that OTUs that are present in a large number of different host species tend to be important for population dynamics within each particular host.

The low connectance, weak, and amensal and/or commensal interactions, together with strong density dependence found in most sponge species, suggest that symbiont communities in the phylum Porifera have stable cores. However, whether these stable cores play a role in the dynamics of remaining OTUs within individual microbiomes, and more importantly, whether this stability guarantees the homoeostasis of host functionality requires further investigation.

### Sponge microbiomes are enriched in specific sequence clusters

Many of the microbes inhabiting sponges have previously been found to fall into monophyletic clusters of ‘sponge-specific' or ‘sponge- and coral-specific' 16S rRNA gene sequences, with these clusters spanning 14 bacterial and archaeal phyla^9^,^12^,^14^,^34^. The ecological and evolutionary significance of these monophyletic clusters remains unclear, yet it is noteworthy that this phenomenon has not been reported outside the phylum Porifera. Over 43% of all sponge-derived sequences from this global sponge analysis were assigned to previously defined monophyletic sponge-specific clusters. However, using deep sequencing and our extensive sampling, 2.7% of seawater sequences and 8.7% of sediment sequences were also assigned to these clusters, demonstrating some clusters are not strictly ‘sponge-specific', but better described as ‘sponge-enriched' (Supplementary Fig. 8)^35^. Importantly, these clusters contain generalists, specialists and opportunists (Fig. 7) indicating that the sponge-specific/enriched microbial sequence clusters have evolved multiple times, either early (that is, core) or late (that is, specialists and opportunists) in the assembly of symbiont communities.

### Host phylogeny and identity structure symbiont communities

**Figure 10:**
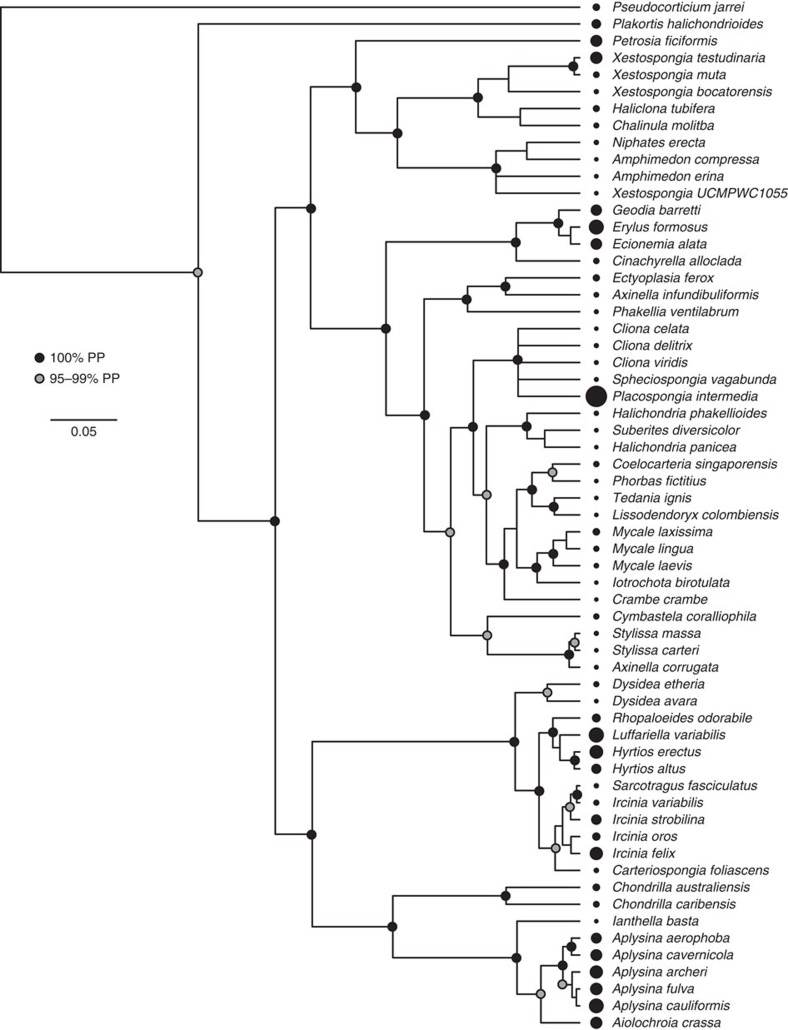
Phylogenetic signal of the inverse Simpson's index (*D*). In this multi-gene phylogeny of host sponge species, 100% Bayesian posterior probabilities (PP) are indicated by black circles at internal nodes, while grey circles indicate 95–99% PP. Nodes with <95% PP are not labelled. Black circles at the tips of the phylogeny are sized in proportion to the mean value of *D* calculated for the symbiotic microbial community associated with each host species. Multiple clades of sponges contain either high (for example, *Aplysina*) or low (for example, *Mycale*) values of *D*.

Environmental and host factors are known to influence the composition of host-associated communities^12^,^36^,^37^; however, the impact of host evolutionary history on the structure and composition of symbiont comunities has only recently been explored^37^. Considering the phylogenetic breadth of sponge species sampled here, we were able to evaluate the relationship between host phylogeny and microbial diversity. Diversity was assessed using the inverse Simpson's index (*D*), while Blomberg's *K* was calculated using the phylosignal function in the R package picante^38^ (Fig. 10; see Methods for details). *K* values of 1 correspond to a random process, values closer to zero correspond to patterns of convergent or random evolution and values >1 indicate phylogenetic conservatism^38^. We observed a significant value of *K* for the inverse Simpson's index (*K*=0.151, *P*=0.027), supporting a significant host evolutionary signal. Pagel's lambda^39^ was calculated to further compare the similarity of covariances among species with the covariances expected, given a random process. The lambda value of 0.216 (AICc=623.3; with *λ* fixed at 0, AICc =627.0) was significantly larger than what would be expected if there was no phylogenetic signal. Combined, these findings indicate a significant signal of convergent evolution in community structure, whereby sponges hosting more diverse communities are more phylogenetically related than expected by chance.

Beta-diversity analysis of symbiont communities (using Bray–Curtis distance) also indicated significant differences among species, with the factor ‘host species' accounting for ∼64% of the observed variation among specimens. A partial Mantel test showed that host phylogeny was significantly correlated with Bray–Curtis distance (*r*=0.442, *R*^2^=0.195, *P*=0.001), as was host identity (*r*=0.706, *R*^2^=0.498, *P*=0.001). Testing for the effect of host phylogeny given host identity greatly reduced the explanatory power of host phylogeny (*r*=0.223, *R*^2^=0.050, *P*=0.001), although host phylogeny still had a significant effect.

Overall, the evolutionary history of the host plays a significant role in structuring the diversity of symbiont communities, but only a minor role in structuring community composition (that is, identity of microbial symbionts), where host identity (reflective of species-level forces) is the more important determinant. Thus, the evolutionary history of the host exerts a significant influence on microbial diversity despite strong selective forces for divergent microbiome composition, which might be critical for niche differentiation among closely related hosts^40^.

### Conclusion

This global microbiome survey of an early-diverging metazoan phylum has revealed that sponges are a reservoir of exceptional microbial diversity and a major contributor to the total microbial diversity found in the world's oceans. Across the Porifera, symbiont communities exhibit little commonality in species composition or structure although a number of emerging properties related to community organization are evident. For instance, sponge symbiont communities are characterized by a predominance of both specialists and generalists (as opposed to opportunists) and the core microbiomes are characterized by generalist symbionts with an under-representation of specialists. These communities represent dynamic systems, with the interacting members featuring all possible ecological interaction types (positive, negative and neutral)^41^. The sign and strength of species interactions among community members has previously been shown to be highly dynamic and contingent on species composition, species densities and the environment^42^. Here we show that the core symbiont communities in sponges are strongly density dependent, have few and weak interactions, low connectance, and amensal and/or commensal interactions indicative of stable core symbionts within the Porifera^30^,^31^,^32^,^33^. Perhaps surprisingly, symbionts that appear to be phylogenetically unique to sponges (that is, having previously been defined as ‘sponge-specific') did not disproportionally contribute to the core microbiome or to any class of symbionts (that is, specialist, generalist or opportunist), indicating that symbiont communities have independently assembled or evolved across the Porifera and that convergent forces have resulted in the analogous community organization and interactions^10^. Although the evolutionary history of the host is undoubtedly a driving force in this process, we show here that host phylogeny primarily impacts the complexity rather than the composition of the symbiont community. These findings further support a model of convergent evolution in symbiont communities across the entire host phylum^10^.

## Methods

### Sampling and sample processing

Samples were taken and processed according to the standard operating procedures to ensure maximum comparability. In brief, at least three different specimens of each sponge species were collected into sterile bags and species identities were confirmed by microscopic examination of morphological characters following established protocols (for example, as reviewed in ref. 43). Specimens were either processed directly or after freezing, depending on logistical constraints of each sampling event. Specimens were cleaned of external growth (for example, barnacles), washed three times with sterile seawater to remove planktonic or loosely associated microorganisms and cut into small pieces from which a random sub-sample of pieces was used for subsequent DNA extraction. Sediment samples were collected under water in close proximity to sponges. Sediments were scooped into sterile containers using sterile spatulas to avoid laboratory contamination. Seawater was drained from the containers on surfacing and prior to freezing. Sponges and sediment samples were immediately frozen and kept on dry ice or at −80 °C until further processing. DNA was extracted from ∼0.25 g of sponge tissue or sediment using the PowerSoil DNA Extraction kit (MoBio) according to the Earth Microbiome Project standard protocols (http://press.igsb.anl.gov/earthmicrobiome/emp-standard-protocols/dna-extraction-protocol/). Microbial communities in seawater were collected by passing 2 l of seawater through 0.2 μm Sterivex filters and DNA was extracted from the filters as previously described^13^. Samples were extracted at laboratories at the Australian Institute of Marine Sciences (Townsville, Australia), the University of Wuerzburg (Germany) or the Nova Southeastern University (Dania Beach, FL, USA) to minimize shipment of frozen specimens. Aliquots of the specimens and DNA were kept at the three locations (and are available on request) and an aliquot of the extracted DNA was shipped to the University of Colorado, Bolder, CO, USA for sequencing of the 16S rRNA gene using standard procedures of the Earth Microbiome Project (http://www.earthmicrobiome.org/emp-standard-protocols/16s/). Briefly, the V4 region of the 16S rRNA gene was amplified using the primer 515f–806rB and sequenced using the HiSeq2500 platform (Illumina)^44^.

### Analysis of sequencing data

We processed Illumina reads in mothur v.1.31.2 (ref. 45). First, quality-filtered, demultiplexed fastq sequences were trimmed according to quality (using the *trim.seqs* command: parameters qwindowaverage=30, qwindowsize=5, maxambig=0, maxhomop=8, minlength=100). Files were reduced to non-identical sequences (*unique.seqs* and *count.seqs*) to minimize computational effort. Non-redundant sequences were aligned (*align.seqs*: flip=t) to a trimmed reference SILVA 102 (ref. 46) bacteria database (*pcr.seqs*: start=11894, end=25319, keepdots=F), which was provided by mothur^47^. Only sequences that were aligned to the expected position were kept (*screen.seqs*: start=1968, end=4411; *filter.seqs*: vertical=T, trump=.). Aligned reads were reduced to non-redundant sequences (*unique.seqs*). Chimeric sequences were detected using Uchime (*chimera.uchime*: dereplicate=t)^48^, and filtered out (*remove.seqs*). Pairwise distances between aligned sequences were calculated (*dist.seqs*: cutoff=0.05) and used for clustering. Prior to clustering, aligned sequences were phylogenetically classified based on the trimmed SILVA database (*classify.seqs*)^49^. Sequences were clustered (*cluster.split*: fasta=, count=, taxonomy=, splitmethod=classify, taxlevel=4, cutoff=0.03, hard=t, method=furthest) and converted to.shared file format (*make.shared*:list=, count=, label=0.03). Finally, OTU representative sequences were retrieved based on the distance among the cluster sequences (*get.oturep*: list=, label=0.03, fasta=, count=) and were further classified based on SILVA, Greengenes (version gg_13_5_99 from May 2013), and RDP taxonomies (*classify.seqs*: fasta=, template=, taxonomy=, cutoff=60)^46^,^50^,^51^. Furthermore, Fastq sequences from additional samples (*n*=340) that were generated at a later time point (using the same sequencing procedure as described above) were processed with the same pipeline. These sequences were integrated into the shared file using QIIME 1.8 (ref. 52), based on their similarity to the OTU representative sequences (*parallel_pick_otus_uclust_ref.py*: --similarity 0.985 --optimal_uclust). Sequences that were not similar to the OTU representative sequences were separately clustered with mothur and integrated into the previous files (.shared and taxonomy files). The integrated OTU table (.shared file) was filtered to remove low-abundance sequences (sequences <0.001% across the whole data set) and chloroplasts (according to SILVA or Greengenes). In addition, counts from seawater-like OTUs (>0.01% across all seawater samples) were removed from sponge samples. File manipulation and processing was carried out with python scripts (http://www.python.org). OTU tables are available in Supplementary Data 4.

### Calculation of community metrics

Rarefaction curves were generated using the R package vegan 2.2-1 (ref. 53). Inter-sample rarefaction curves were generated by mothur (*rarefaction.shared*). Distances of the samples in a group (sponge species, seawater or sediment) and their respective group centroids were calculated based on Bray–Curtis distances by the function *betadisper* from the vegan package in R (ref. 53). Richness indicators (Chao, Ace, Sobs) were also calculated with vegan. Nonmetric multidimensional scaling was calculated with vegan package based on Hellinger transformed OTU counts^54^. Taxonomic profiles were obtained based on Greengenes, which provided more phylum-level assignments than the SILVA or RDP databases. Briefly, percentage OTU counts were averaged by species/environment with the R package analogue 0.16-0 (ref. 55). Phylum percentages were calculated by summing averaged OTU percentages. Bray–Curtis dissimilarities were calculated and heatmap was obtained using the package pheatmap 1.0.7 (ref. 56).

### Sponge-bacteria bipartite network analysis

A bipartite interaction network was constructed using the presence of specific OTUs within each of the sponge species in the data set. OTUs were considered part of the network only if they were found in at least 25 distinct samples from the whole data set. In this bipartite host–microbe interaction network, nodes represent sponge species (on one side) and OTUs (on the other); and links among them represent the presence of an OTU in the microbial community of the sponges to which it is linked. The network was constructed using a software script developed in R using the package igraph 0.7.1 (ref. 57) and interrogated using statistical tools to describe its properties.

The degree distribution of sponges and OTUs was analysed to assess the heterogeneity of the network in terms of node connectivity. Degree distributions depict the statistical probability distribution of finding nodes with a certain degree (number of other nodes it is connected to). A variant of the degree distribution was employed: the cumulative degree distribution, which has the same probability distribution, but shows the probability of finding nodes with that degree or less. These probability distributions (one for the OTUs and another for the sponges) were obtained using the *cumsum* function in R (ref. 58). In addition, we fitted truncated power law and exponential functions to the OTUs and sponges cumulative degree distributions, respectively. This was achieved using the non-linear least squares (nls) function provided by R. This analysis reveals the pattern of connectivity between sponges and OTUs and facilitates determination of the balance between generalist and specialist species. The thresholds for specialism and generalism were chosen arbitrarily, but following basic requirements for this type of network analysis. First, neither of the groups contains the parameter that provides the characteristic scale at which the exponential cutoff occurs in the truncated power-law distribution. In our case, this value is 7.44 (Pc(*k*)=*k*^−0.32^ × *e*^−(*k*/7.44)^), so specialists need to have a number of links below that threshold, and generalist species should have a number of links that is several times this number—for this purpose we selected seven times this number. Second, the average number of links in the specialist and generalist groups should be very different from the mean number of links in the network, and the difference in this ratio (mean group/mean network) should be similar for both groups. The mean number of network links is 12.13, the mean number of links for specialists is 2.5 and for generalists is 60, with specialists thus having a mean number of links ∼4.47 times smaller than the average, and generalists 4.66 times larger than the mean.

The relationship between the fraction of samples within which a given OTU is found for a particular sponge species versus the total number of sponges in which that OTU was found (degree of the OTU in the network) was also assessed. This was achieved by obtaining the fraction of sponge samples in which a given OTU was found out of all the samples available for the sponge hosts to which that OTU is connected in the host–microbe network. This information was plotted against the degree of the OTU. To better visualize this relationship a smoothing spline was fitted using the *smooth.spline* function provided by R. This relationship is used to analyse the true shape of specialisation versus generalism in ecological networks.

### Bacteria–bacteria network analysis

For the bacteria–bacteria network analysis, we focused on host species with more than 47 individual replicates. If more than 47 replicates were available, we randomly subsampled 47 replicates. Cores were created for each host species by extracting OTUs occurring in at least 85% of the 47 replicates and were further filtered by removing OTUs with a relative abundance <0.01. This was done by using *filter.shared(minpercentsamples=85, minpercent=1, makerare=f)* in mothur v.1.31.2. The statistical model developed in ref. 27 for inferring interactions from temporal series data was adapted to substitute time for space such that spatial replicates for each host were used rather than temporal samples. If we denote *n*_*i,m*_ as the natural logarithm of *N*_*i,m*_, then on a natural logarithmic scale we have the number of sequences of core OTU *i* in replicate *m* within any given host species described by


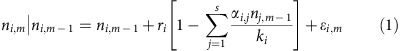


where *r*_*i*_ and *k*_*i*_ represent the intrinsic growth rate and the carrying capacity of OTU *i*, respectively, *α*_*i,j*_ represent the interaction coefficient between OTU *i* and *j* and expresses the per capita effect of OTU *j* on the growth rate of OTU *j* from replicate *m*−1 to replicate *m*. Finally, *ɛ*_*i,m*_ represents the effect of unexplained, latent, stochastic noise on the population dynamics of species *i*. The time-series is modelled using a Poisson distribution, where *y*_*i,m*_ denotes the observed number of sequences of OTU *i* in replicate *m*


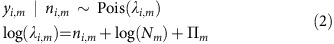


where log(*N*_*m*_) and Π_*m*_ represent offsets to model rates instead of counts. Both correspond to the total abundance in replicate *m*, but the latter is treated as a random normal variable with a mean of zero and a s.d. of 100.

*r*_*i*_ is assumed to be ∼*N*(0, 10), while *k*_*i*_*∼Exp*(1) to limit *k*_*i*_ to positive values.

The total variance *V*_*i*_ of individual OTU abundances can be decomposed into additive contributions from inter-specific interactions, density-dependence and stochasticity, respectively,


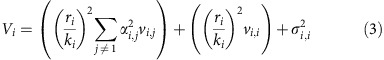


where *v*_*i*,*i*_ is the stationary variance for *n*_*i*_. From this, the proportion of variation attributed to each source of variability can be calculated (see ref. 27 for details).

We used Gibbs variable selection method^59^ to constrain the model to only use inter-specific interaction coefficients, *α*_*i,j*_, for which there are strong support in the data. This is achieved by introducing a binary indicator variable *γ*_*i*,*j*_ for *i*≠*j*, and assuming *γ*_*i*,*j*_∼Bernoulli(*P*), such that *γ*_*i*,*j*_=1 when species *j* is included in the dynamics of species *i*, and *γ*_*i*,*j*_=0 otherwise. Where there is low support for the inter-specific interaction in the data, *γ*_*i*,*j*_=0, and the interaction is excluded from the model. When *γ*_*i*,*j*_=1, *α*_*i,j*_ is freely estimated from the data. A *P* of 0.1 was selected as we do not expect more than 10% of all possible inter-specific interactions to be realized.

We use Markov chain Monte Carlo simulation methods in R using the runjags package^60^ to sample from the joint posterior distribution of the model parameters. We ran 10 independent chains with dispersed initial values for 5e6 iterations, discarding the first 2e6 samples of each chain as burn-in and thinned the remainder to every 50th sample. We evaluated convergence of model parameters by visually inspecting trace and density plots. In addition, to ensure good mixing of *α*_*i,j*_ we calculated the number of jumps *γ*_*i,j*_ did between its two states (0 and 1).

Finally, to build the representative networks, we analysed the interaction and sign structure of the posterior distribution for the interaction coefficient *α*_*i,j*_. *α*_*i*,*j*_ is a full probability distribution, hence it contains the probability of OTU *j* having a non-zero per capita effect on the growth of OTU *i* (interaction strength), and vice versa. Using all information in *α*_*i,j*_, we constructed a representative network for each host species as a mean of visualizing the most ‘credible' network structure. This was done by mapping the posterior average number of links onto *α*_*i,j*_, and in doing so, extracting the links with the highest probability of non-zero interactions. This was done by custom-written R scripts. As a way of validating the structure of each representative network, we compared the connectance of each representative network with the posterior average connectance for *α*_*i,j*_ for each host species. The representative networks was plotted using the igraph package^57^ in R.

### Identification of sponge-specific and sponge/coral-specific clusters

A representative sequence from each OTU was taxonomically assigned using a BLAST^61^ search against a curated ARB-SILVA database containing 178 previously identified sponge-specific clusters (SC) and 32 sponge/coral-specific clusters (SCC)^14^. For each BLAST search, the 10 best hits were aligned to determine sequence similarities. The most similar OTU sequence to the respective reference sequence within the database was then assigned (or otherwise) to an SC or SCC based on the application of a 75% similarity threshold (that is, a sequence read was only assigned to a cluster if it was more similar to the members of that cluster than to sequences outside the cluster and its similarity to the most similar sequence within that cluster was above 75%). In cases where the assignment of the most similar sequences was inconsistent, a majority rule was applied, and the OTU sequence was only assigned to an SC or SCC if at least 60% of the reference sequences were affiliated with this cluster.

### Phylogenetic analysis of host species and correlation with symbiont communities

Our phylogenetic analysis considered 61 sponge species for which at least one of three gene sequences (small subunit of nuclear ribosomal RNA [*18S*], large subunit of nuclear ribosomal RNA [*28S*] or mitochondrial cytochrome oxidase subunit 1 [*cox1*]) could be obtained from GenBank. For 39 of the 61 species (64%), sponge gene sequences were also obtained from at least one identical specimen collected for the current study. The biologists who collected specimens were very familiar with sponge taxonomy and identification, and we intentionally focused our study on easily recognized and common taxa to avoid misidentification. Some genetic markers used for sponge taxonomy are easier to sequence than others and here we used all available data for the most frequently used markers for sponge molecular systematics.

For each gene, sequences were aligned using the default options of MAFFT 7.017 (ref. 62). Each alignment was analysed using the Gblocks Server^63^ to eliminate non-conserved regions; the resulting three alignments were then concatenated using the Geneious software (version 6.1.8, Biomatters Limited). The phylogeny was constructed with MrBayes version 3.2.1 (ref. 64), using the computational resources provided by CIPRES^65^. Within MrBayes, five partitions (*18S*, *28S* and the three codon positions of *cox1*) were specified and separate general time reversible models of evolution for each partition were estimated, incorporating a gamma distribution of substitution rates among sites and a proportion of invariant sites (GTR+I+G) as suggested by ref. 66. The Homoscleromorpha *Pseudocorticium jarrei* and *Plakortis halichondrioides* (the only non-Demospongiae sponges of the taxon set) were constrained as an outgroup and the independent gamma rates relaxed-clock model with a birth–death process was implemented. The phylogenetic analysis included three parallel runs of 10 million generations, each utilizing four Markov chains and sampling every 100 generations. At the end of the runs, we assessed convergence by the average s.d. of split frequencies, which was 0.03, and the potential scale reduction factors of all parameters, which ranged from 1.00 to 1.01. Following a burn-in of 25%, the trees sampled by each of the three runs were summarized into a consensus tree. The diversity of OTUs associated with each host species was evaluated by calculating OTU richness, the Shannon index of diversity, and the inverse Simpson index of diversity. All indices demonstrated clear differences among sponge species (*P*<0.001). Beta-diversity analysis was conducted by calculating the Bray–Curtis distance among specimens, and testing for host species differences in this distance using the function adonis in the R package vegan^53^.

### Data availability

Processed sequences can be downloaded from the following portal: http://qiita.microbio.me (Study ID 10346). Sequence data have been deposited in the BioProject database under accession code PRJEB11983 and in the European Nucleotide Archive under accession code ERP013416. The authors declare that all other data supporting the findings of this study are available within the article and its Supplementary Information files.

## Additional information

**How to cite this article**: Thomas, T. *et al.* Diversity, structure and convergent evolution of the global sponge microbiome. *Nat. Commun.* 7:11870 doi: 10.1038/ncomms11870 (2016).

## Supplementary Material

Supplementary Information Supplementary Figures 1-8 and Supplementary Tables 1-2

Supplementary Data 1 Diversity and richness of sponge species.

Supplementary Data 2 Metadata for sponge samples analysed in the current study.

Supplementary Data 3 Representative sequences and taxonomy of OTUs.

Supplementary Data 4 OTU cluster and their counts across samples.
