# Supplementary material for: Diversity, structure and convergent evolution of the global sponge microbiome
Source: Nat Commun. 2016 Jun 16;7:11870. doi: 10.1038/ncomms11870 (PMC4912640; doi:10.1038/ncomms11870)
Supplement: Supplementary Information — Supplementary Figures 1-8 and Supplementary Tables 1-2 [file ncomms11870-s1.pdf]

Supplementary Information

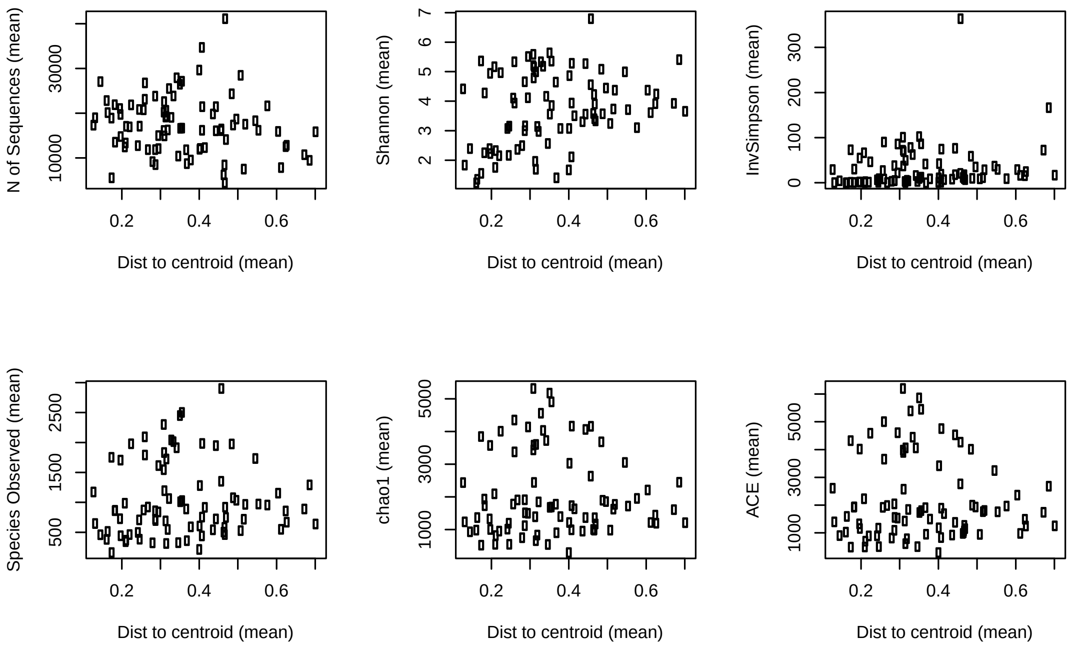

Supplementary Figure 1: Scatterplot of the intraspecific community dissimilarity (measured as distance of samples to group centroids) and various alpha-diversity measurements for 16S rRNA gene diversity of symbionts in individual sponge species.

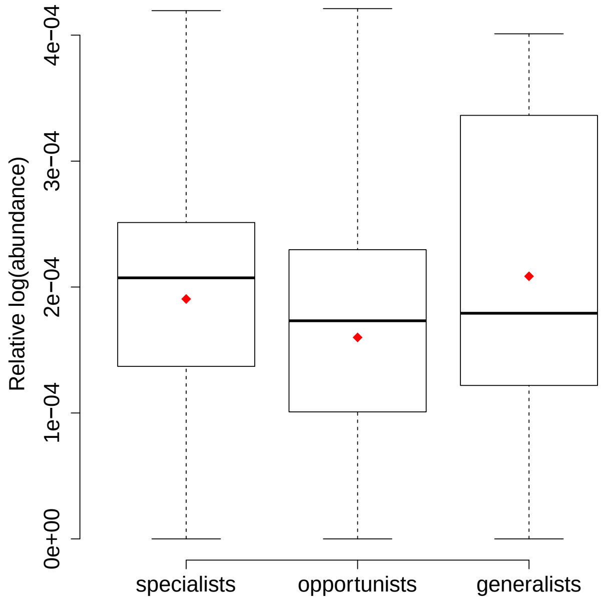

Supplementary Figure 2: Relative log abundances of OTUs across the entire sponge-bacteria network classified according to their connectivity pattern. Specialists = OTUs present in less than 5 different sponge species; opportunists = OTUs present in between 5 and 50 different sponge species; and generalists = OTUs present in more than 50 sponge species. Red diamonds show the mean value per group. In the boxes, central rectangles span the first quartile to the third quartile (the interquartile range). Segments inside rectangles show the median and "whiskers" above and below the boxes show the locations of the minimum and maximum values.

a. *Carteriospongia foliascens*

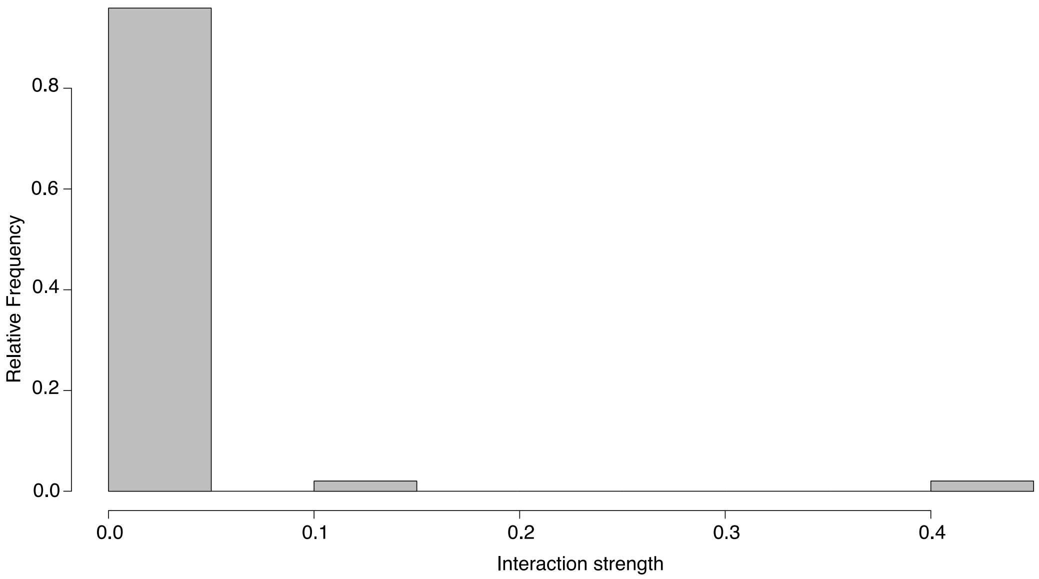

b. *Cliona delitrix*

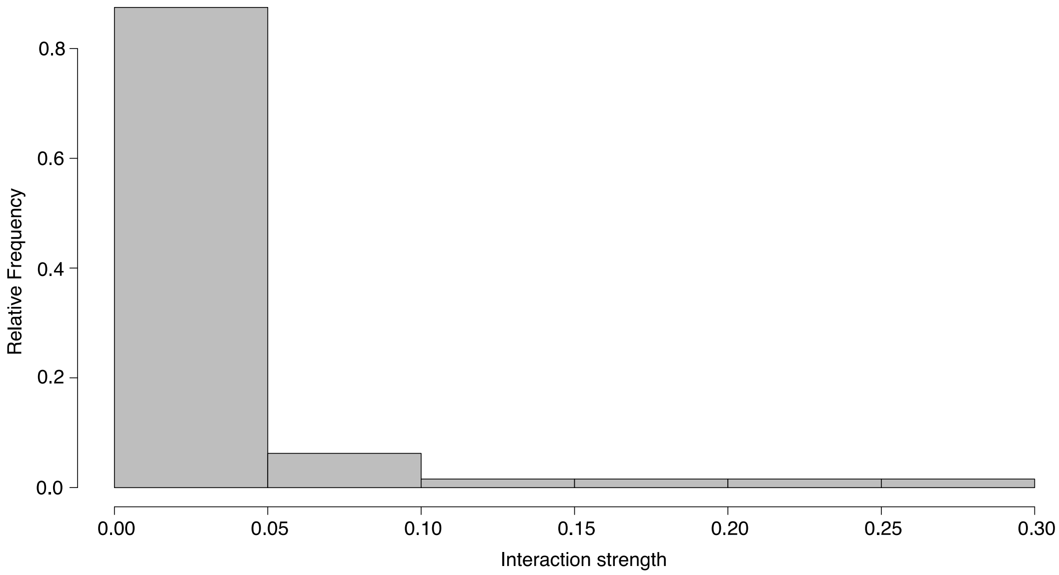

c. *Ircinia oros*

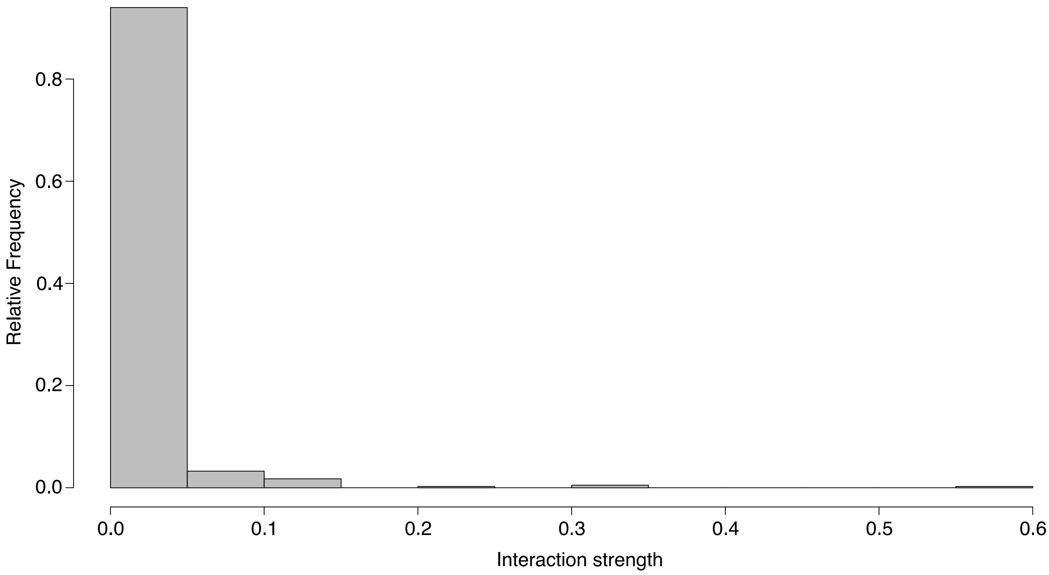

d. *Ircinia variabilis*

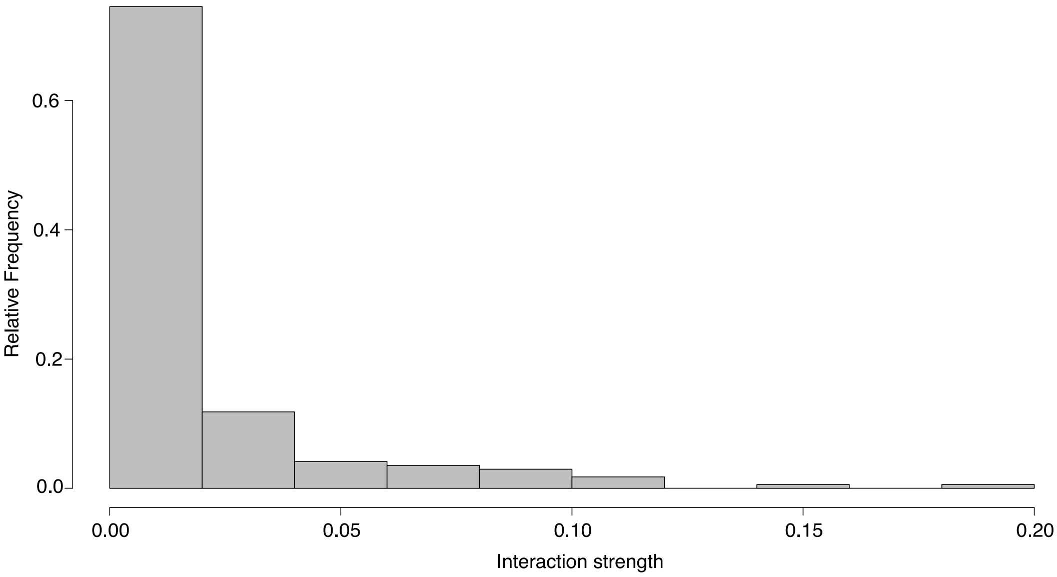

e. *Sarcotragus fasciculatus*

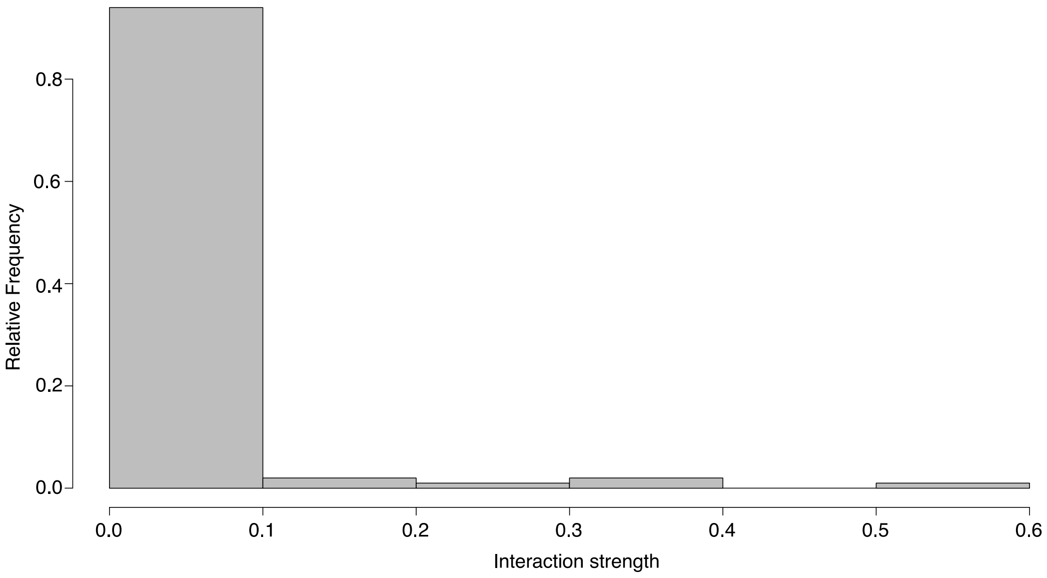

Supplementary Figure 3: Frequency histogram of interaction strengths for core microbiome of different sponge species. Interaction strength is calculated from the posterior distribution of the interaction coefficient alpha (Equation 1 in the main text). Note that across host species a skewed distribution is observed characterized by many weak and a few strong interactions.

a. *Carteriospongia foliascens*

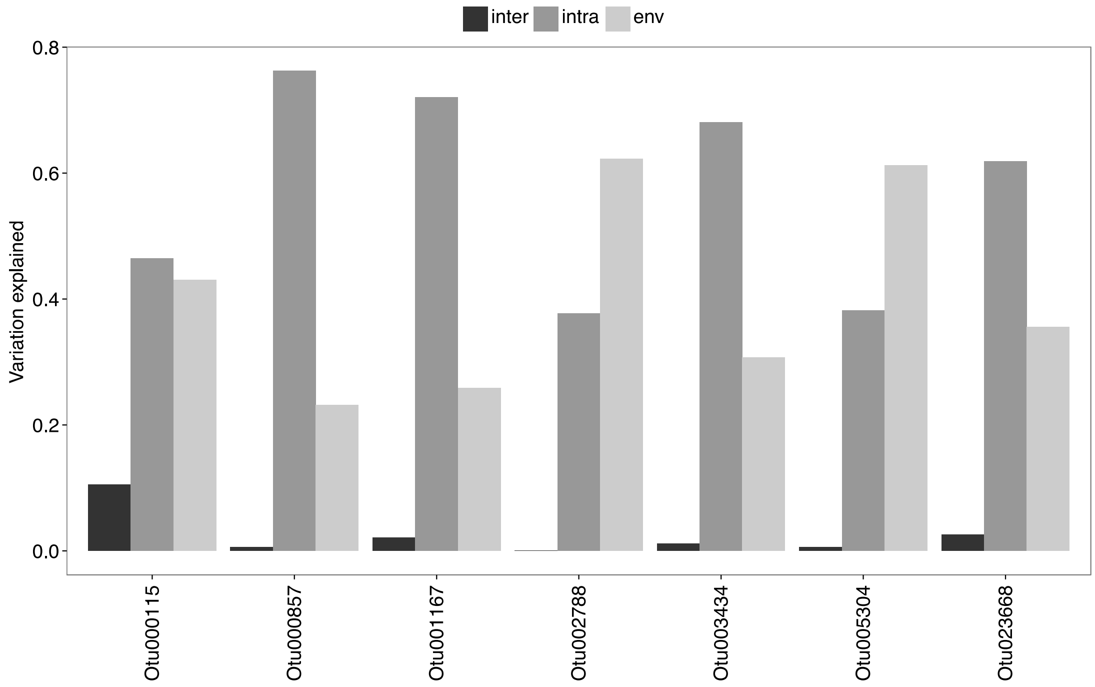

b. *Cliona delitrix*

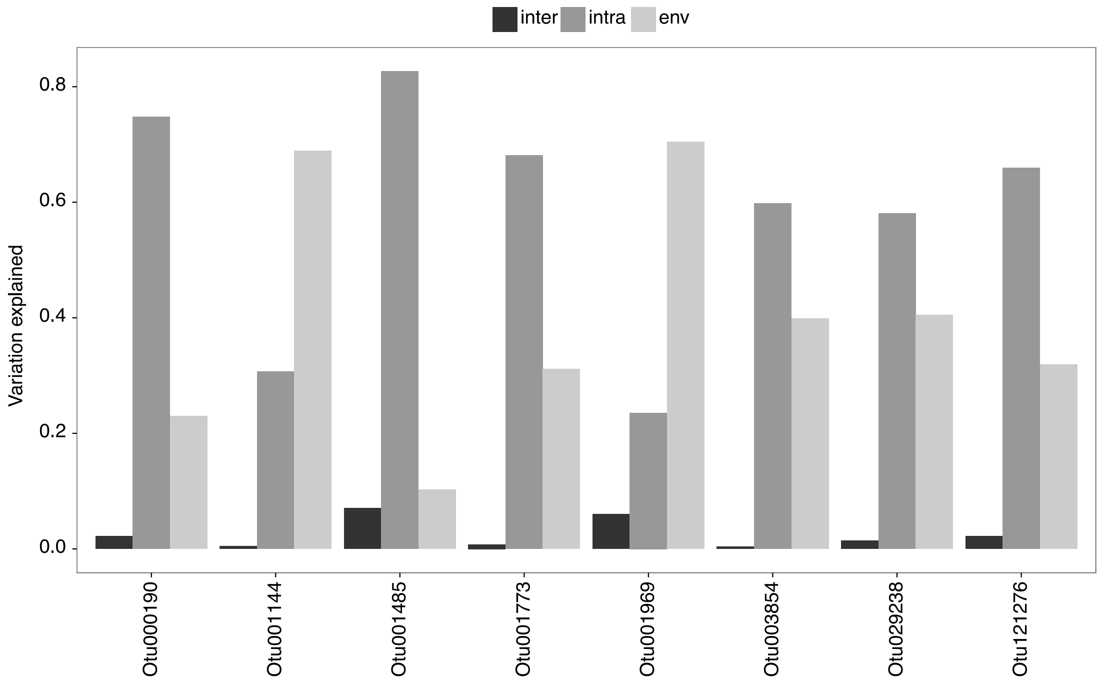

c. *Ircinia oros*

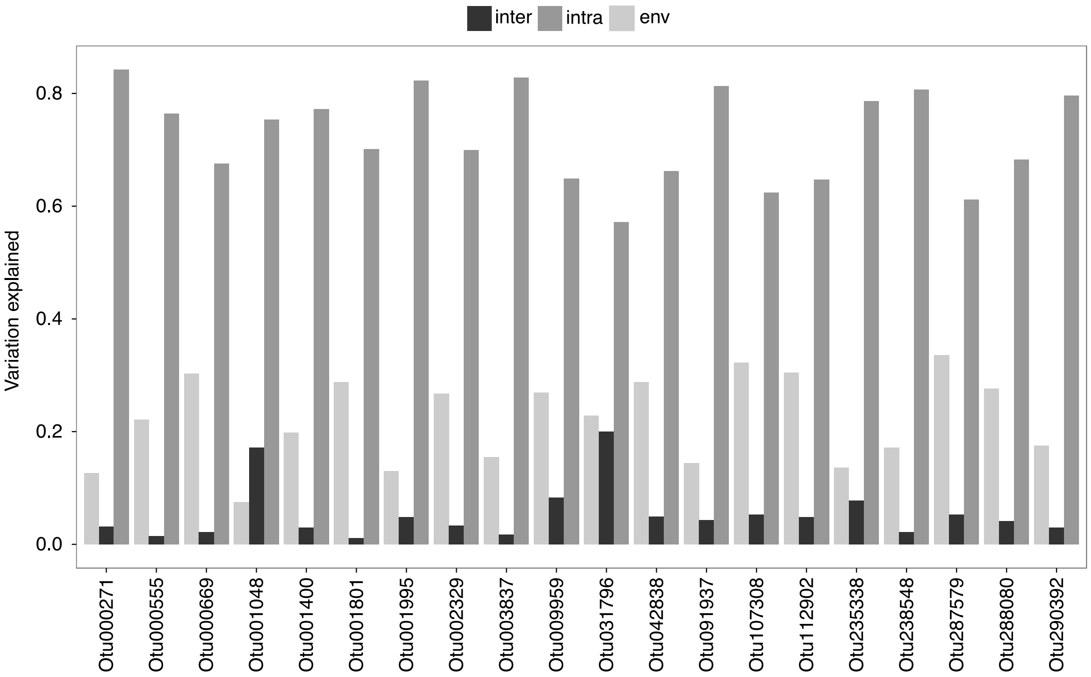

d. *Ircinia variabilis*

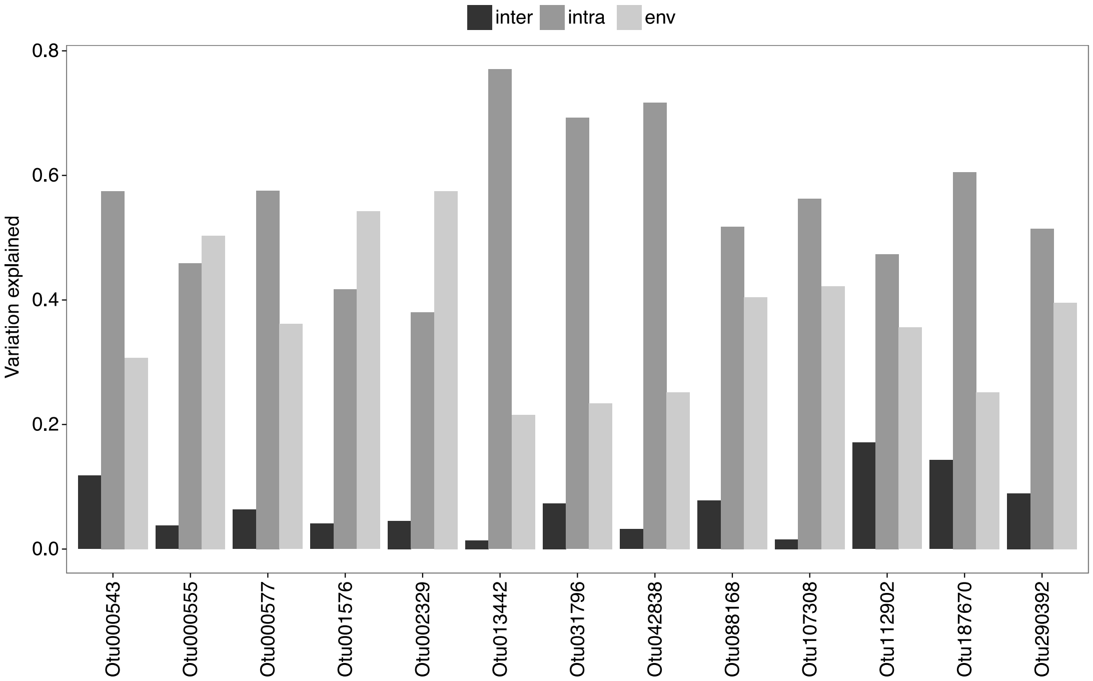

e. *Sarcotragus fasciculatus*

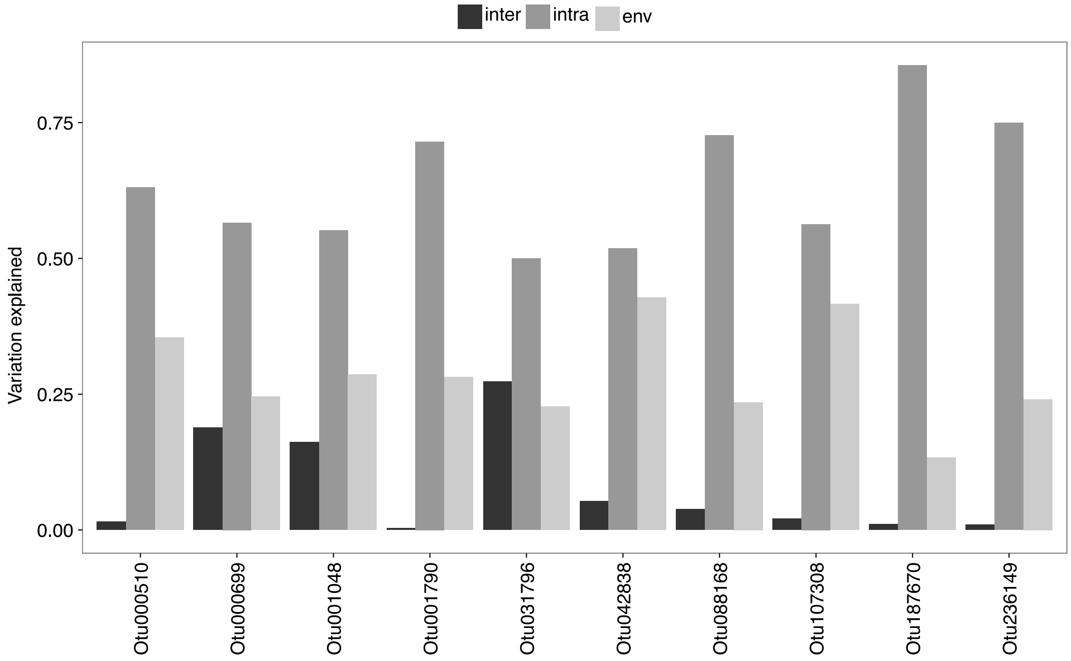

Supplementary Figure 4: Variation partitioning per OTU within each core microbiome following equation 3 in the main text. The y-axis shows the variation explained by interspecific interactions (black), density dependence (dark grey) and environmental stochasticity (light grey). The x-axis shows the different OTUs present in the different core microbiomes. Across hosts, density dependence explains the largest part of the variation followed by environmental stochasticity and inter-specific interactions (for total values; see Supplementary Table 1).

a. *Carteriospongia foliascens*

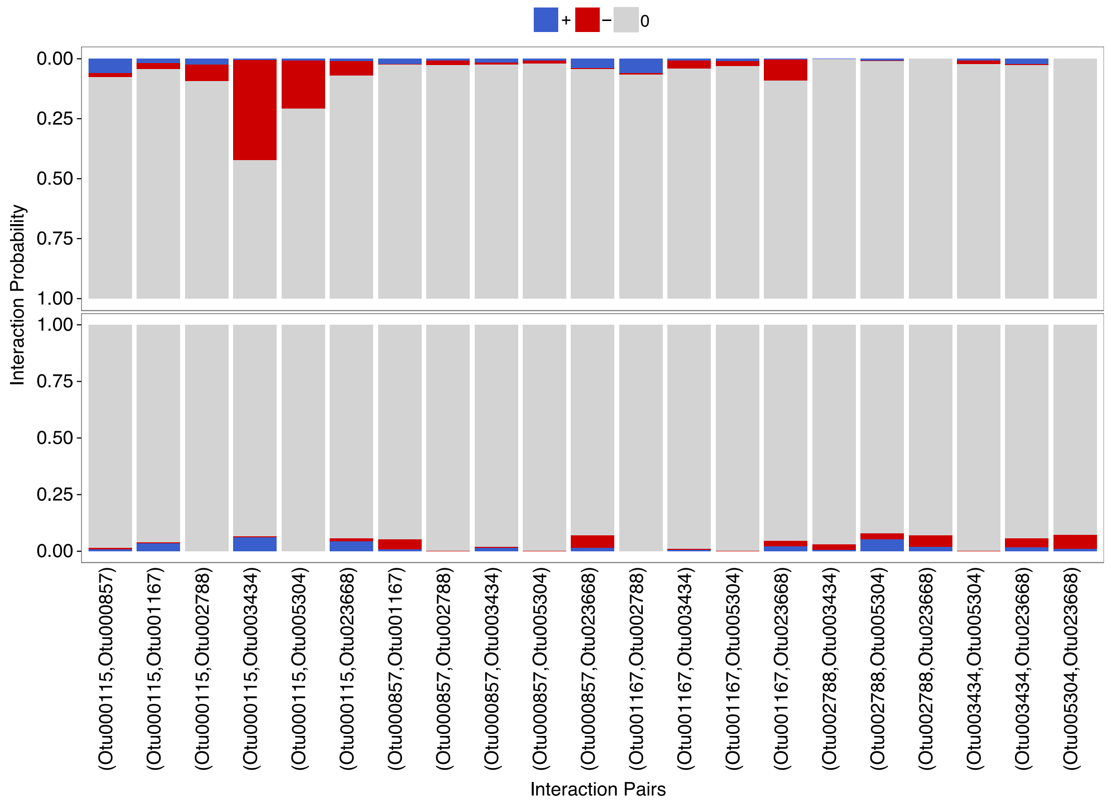

b. *Cliona delitrix*

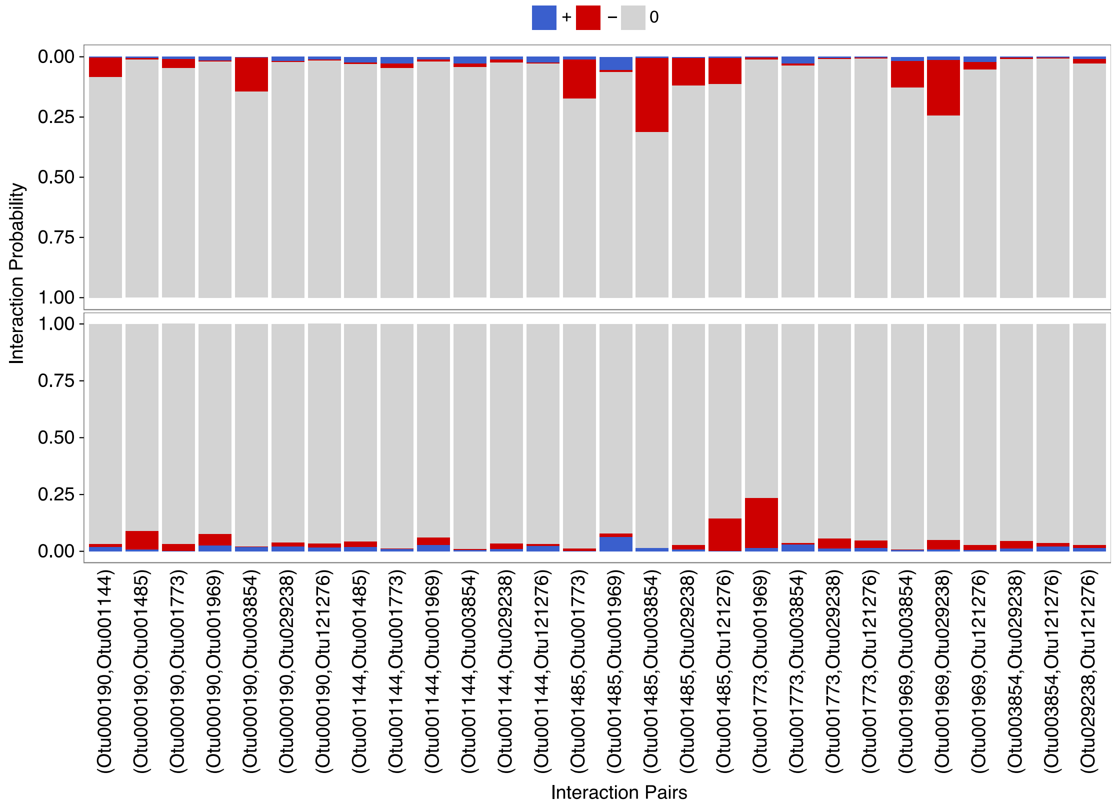

c. *Ircinia oros*

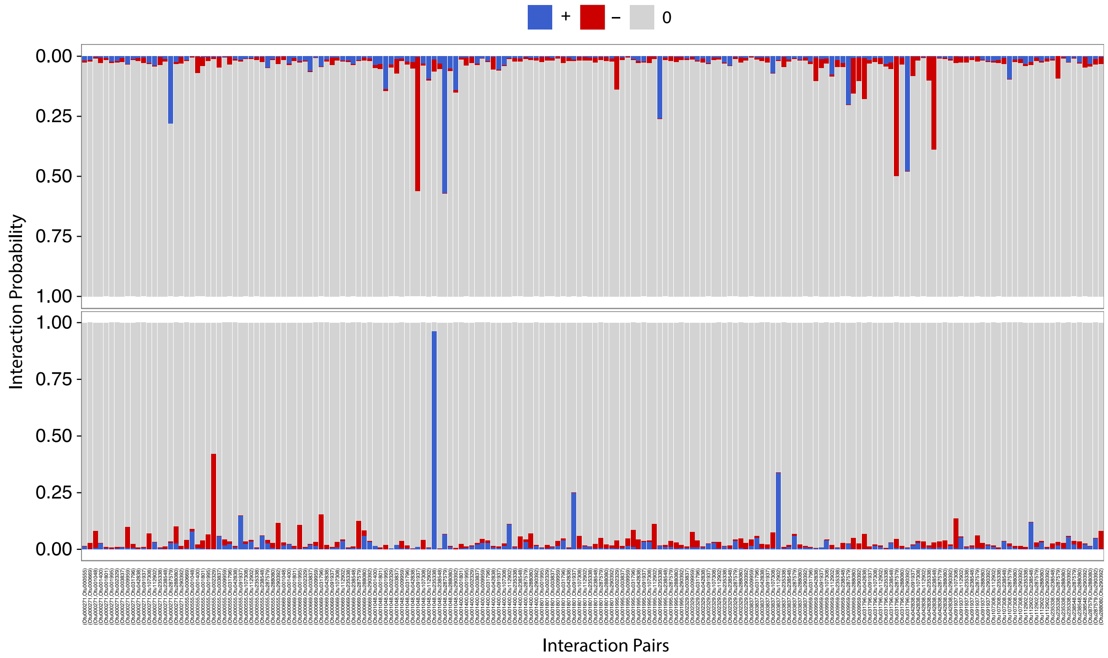

d. *Ircinia variabilis*

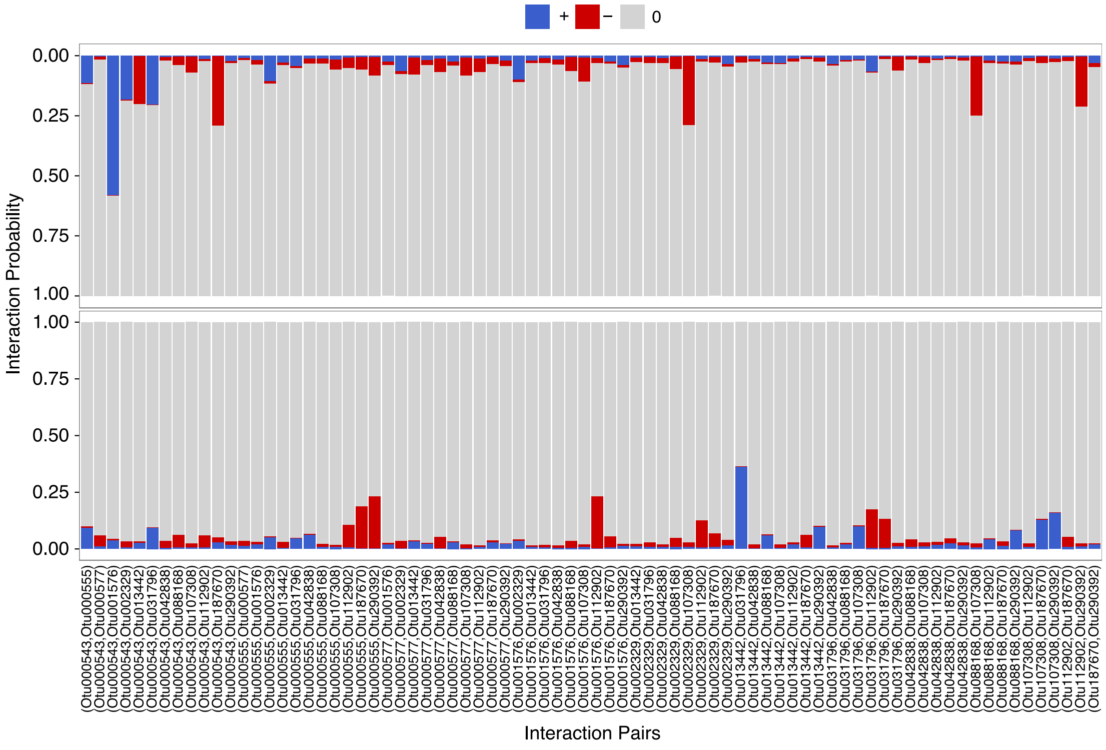

e. *Sarcotragus fasciculatus*

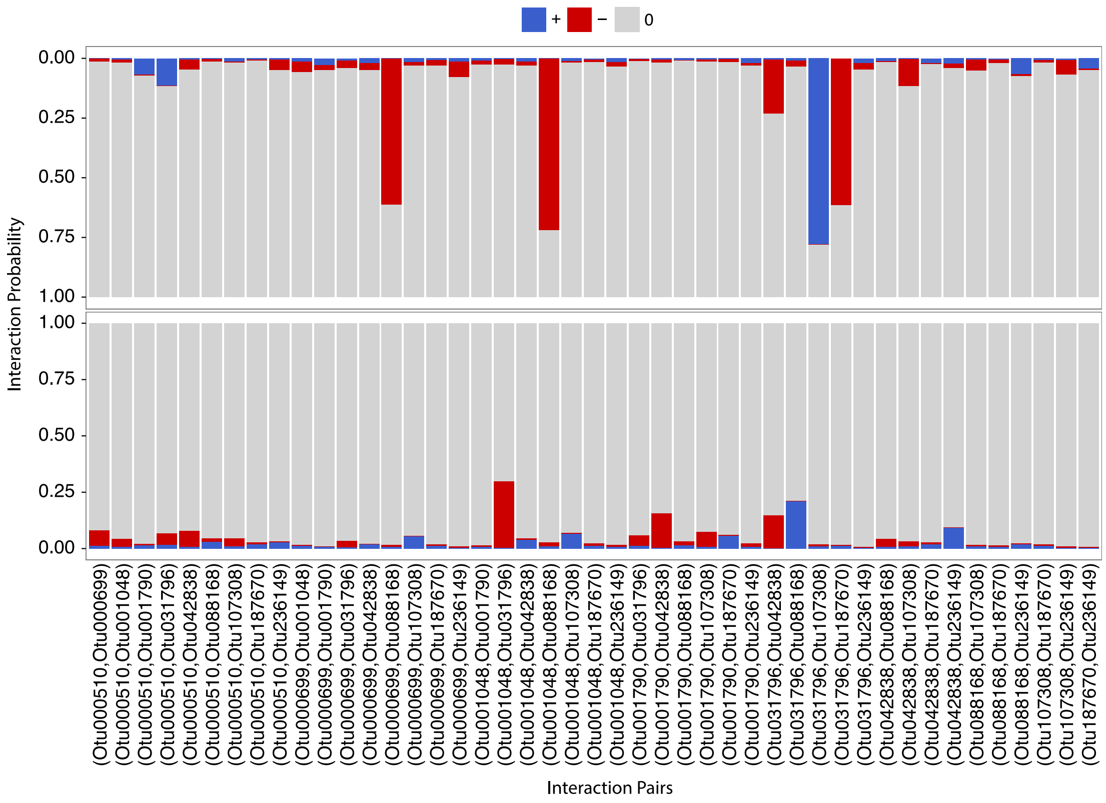

Supplementary Figure 5: The probability of all possible interactions within each core microbiome. This is calculated from the posterior distribution of the interaction coefficient alpha (Equation 1 in the main text). The top y-axis shows the probability for *species j* to interact with *species i* and the bottom y-axis shows the vice versa. The x-axis shows all the possible OTU pairwise interactions. Blue colour represents the probability for a positive interaction, red for a negative interaction, and grey shows the remaining zero probability. Note that an interaction can have a certain probability of being both positive and negative. Overall, there is a mixture of positive and negative interactions, with an increasing fraction of positive interactions as the core microbiome increase in size. Also, a ‘high’ probability interaction (regardless of sign) by one partner is usually met by a ‘weak’ probability interaction by the second partner, corresponding to commensalism and/or amensalism.

a. *Carteriospongia foliascens*

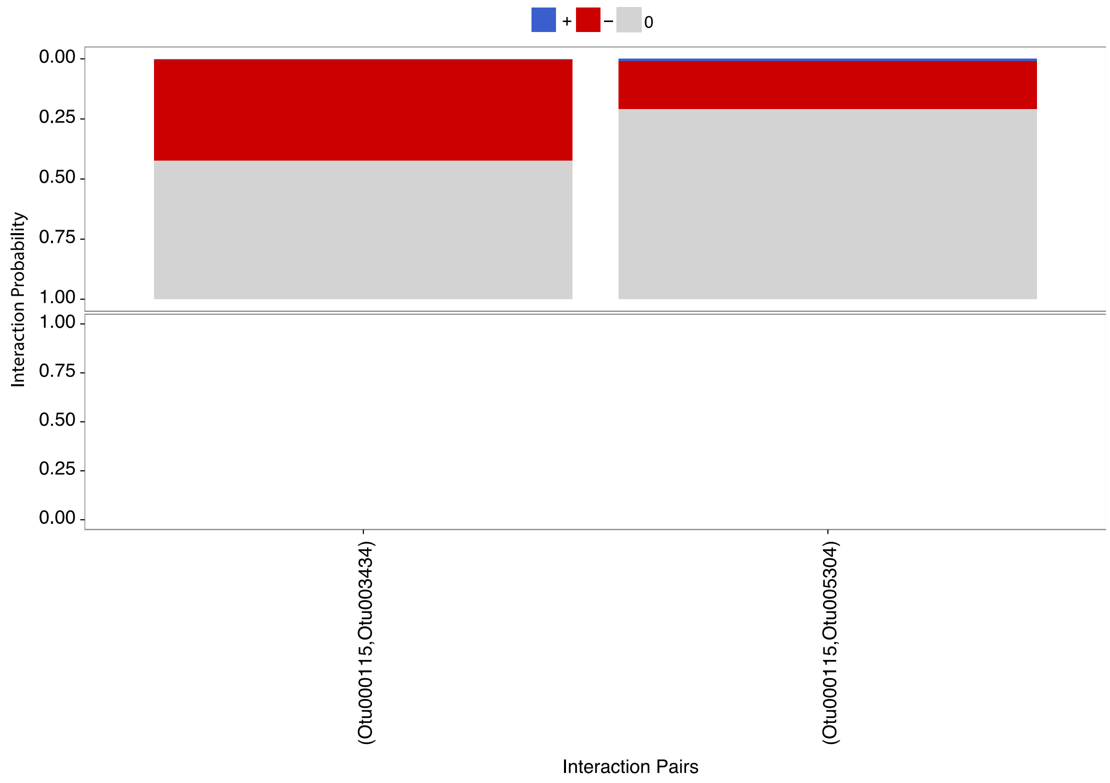

b. *Cliona delitrix*

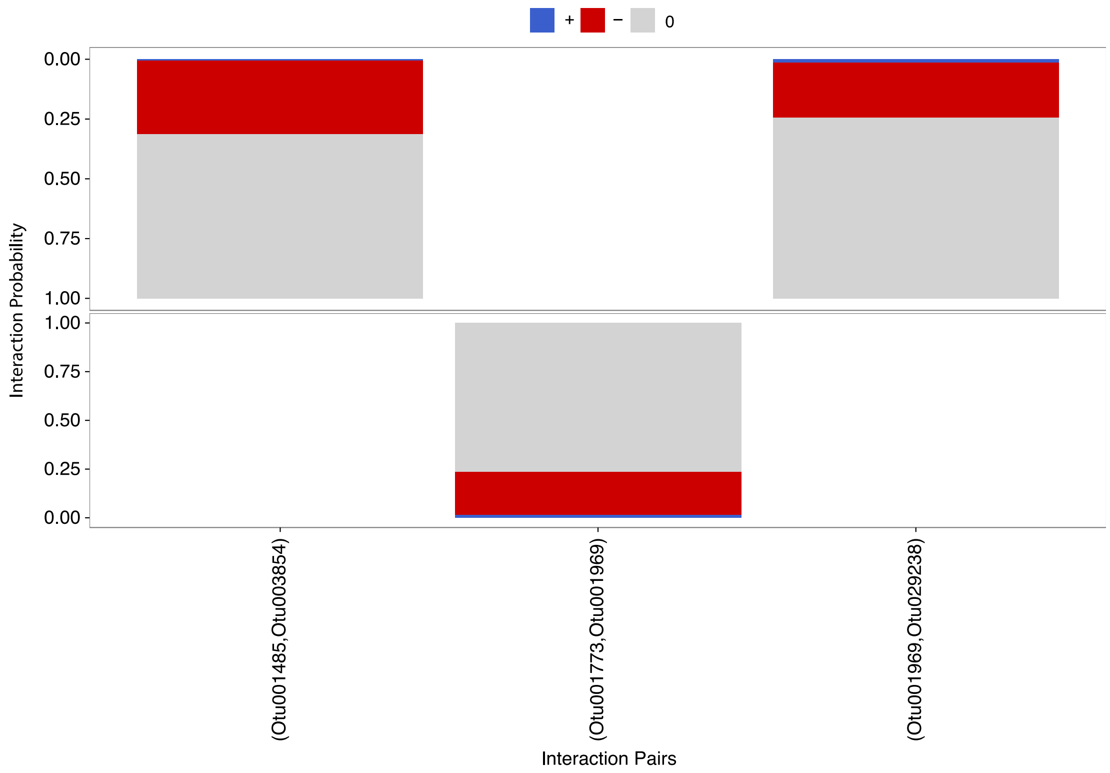

c. *Ircinia oros*

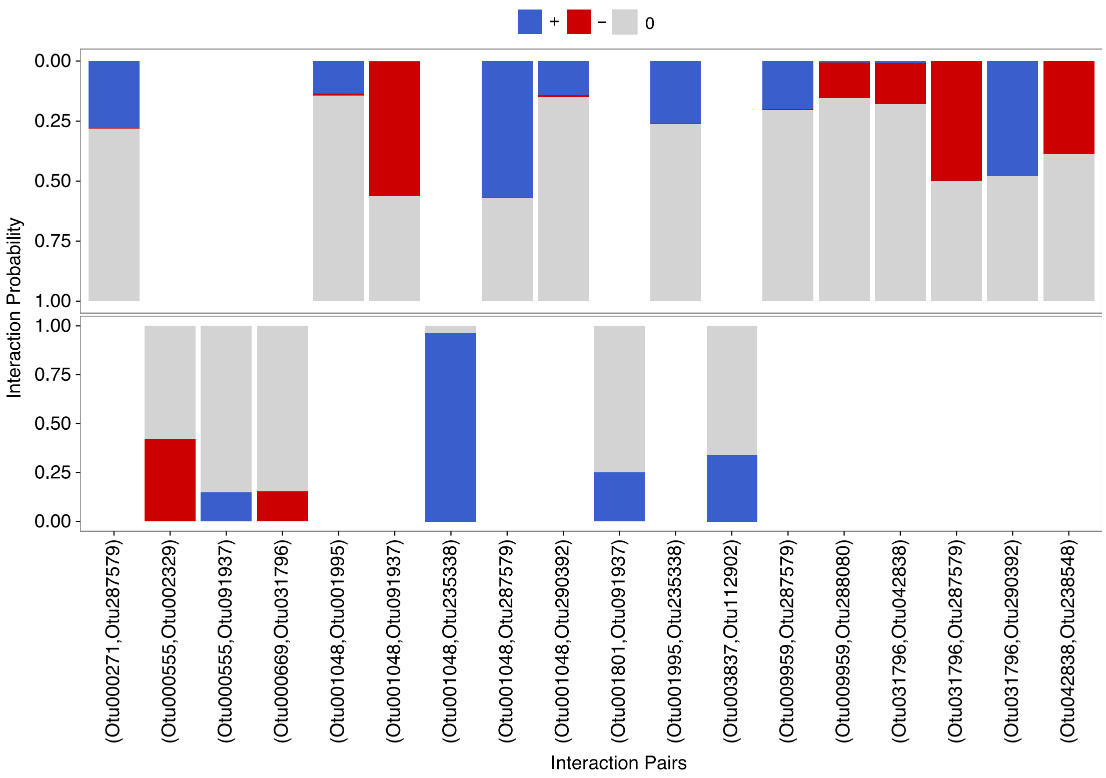

d. *Ircinia variabilis*

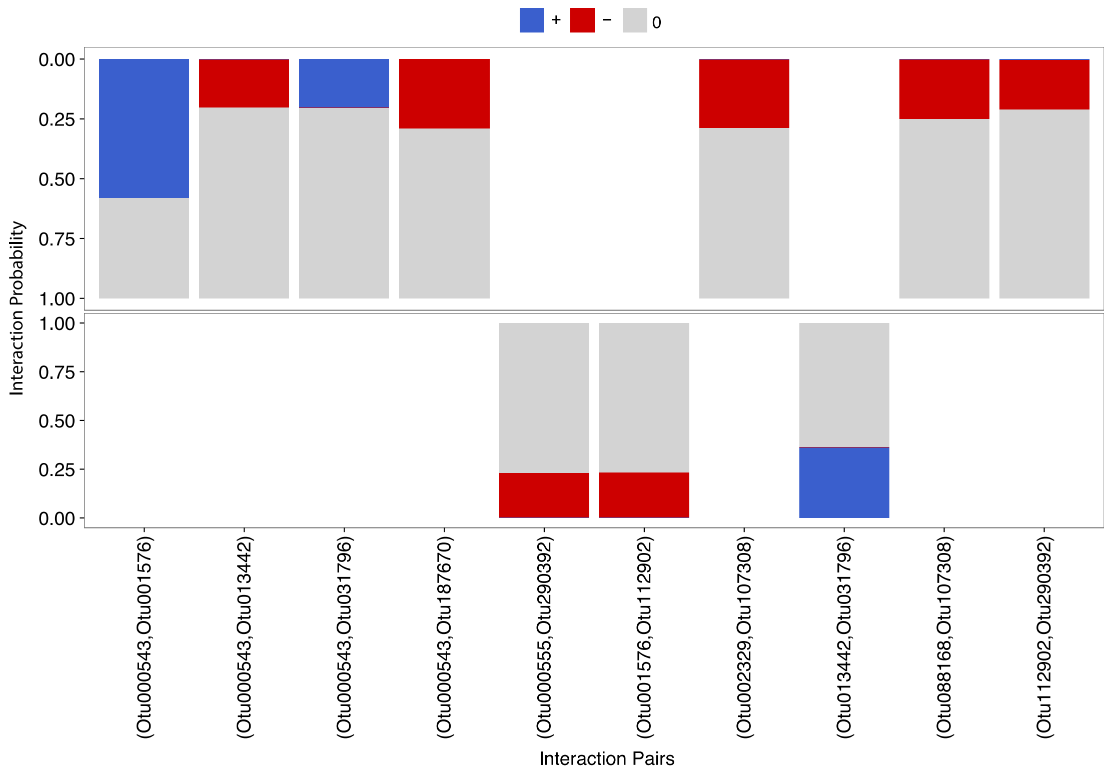

e. *Sarcotragus fasciculatus*

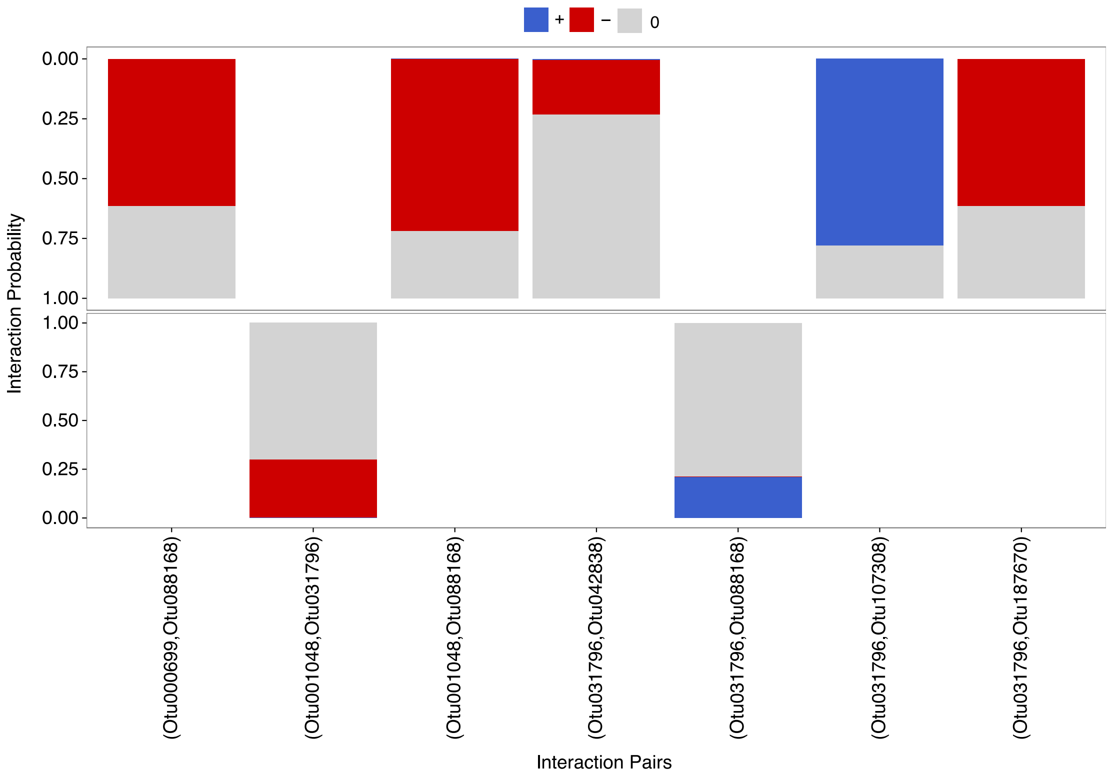

Supplementary Figure 6: Shows a subset of the most probable links shown in Supplementary Figure 4. The number of links corresponds to the posterior average number of links (see Supplementary Table 2) with the highest probability for each core microbiome. The top and bottom y-axes show the probability for *species j* to interact with *species i* and vice versa. The x-axis shows the subset of OTU interaction pairs with the highest probability. Blue colour represents the probability for a positive interaction, red negative and grey shows the remaining zero probability. Note that an interaction can have a certain probability of being both positive and negative.

a. *Carteriospongia foliascens*

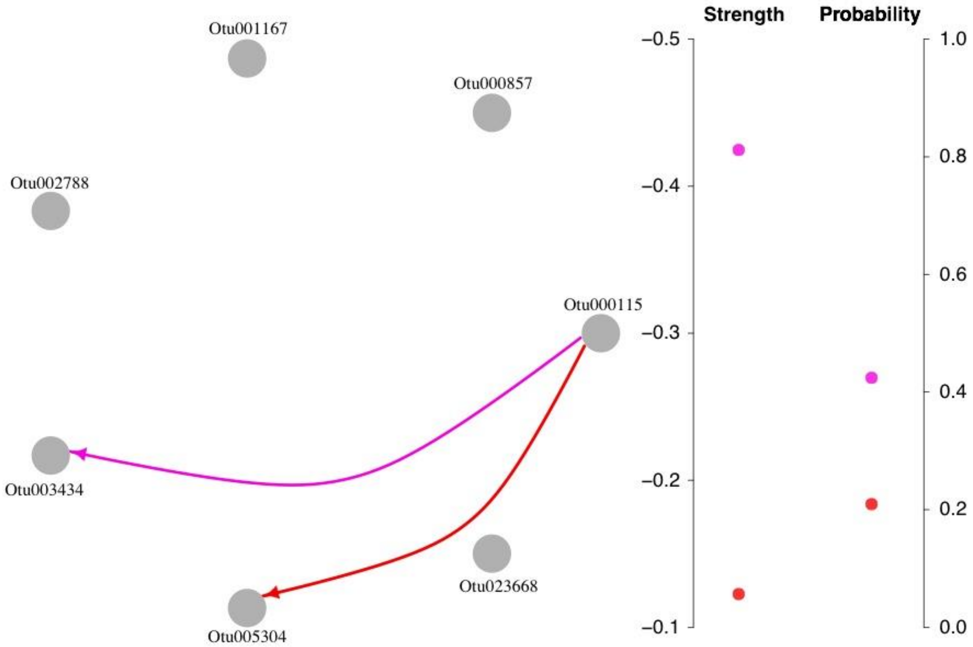

b. *Cliona delitrix*

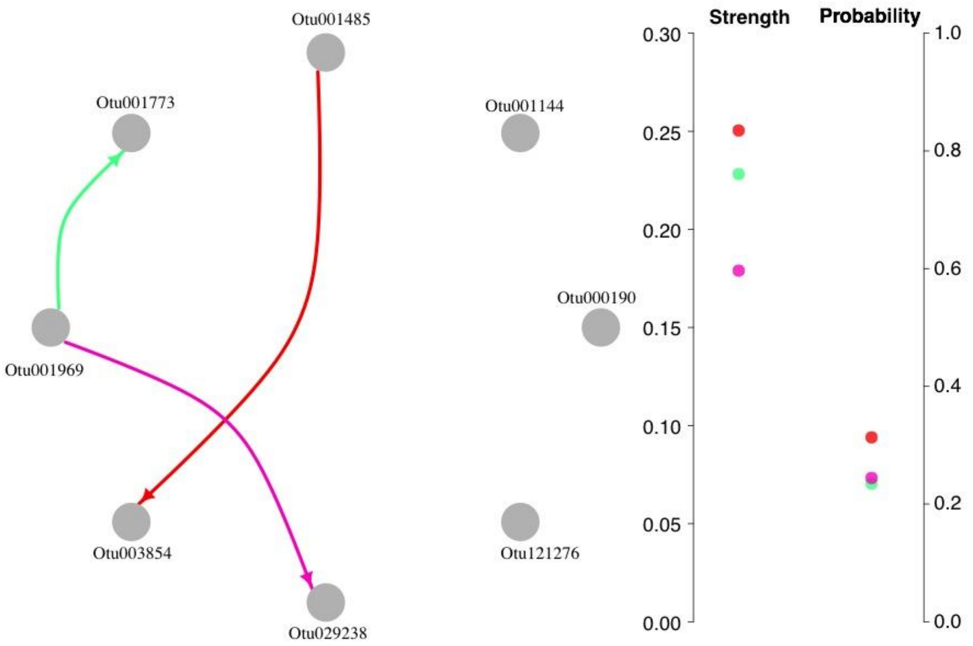

c. *Ircinia oros*

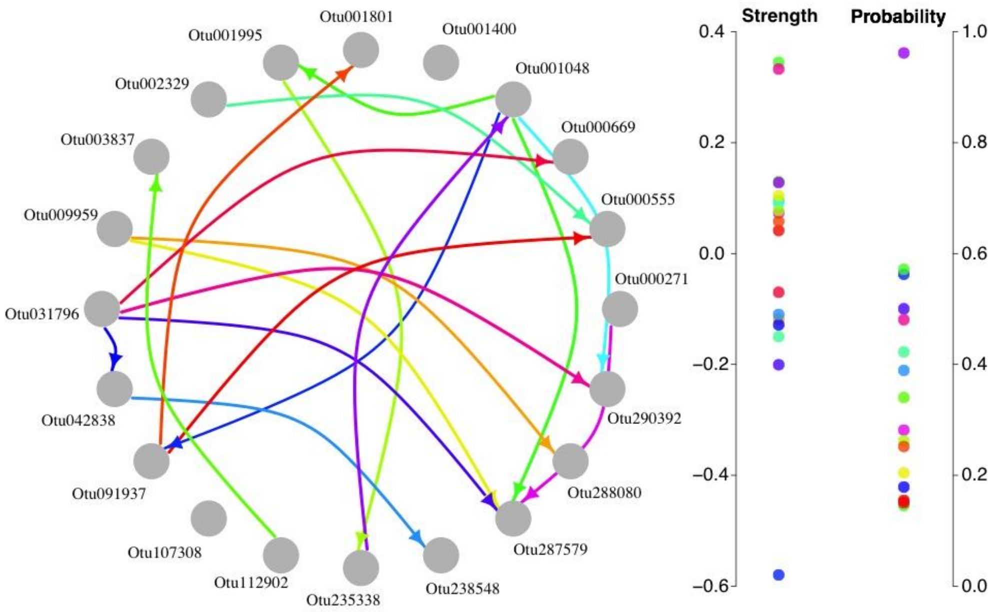

d. *Ircinia variabilis*

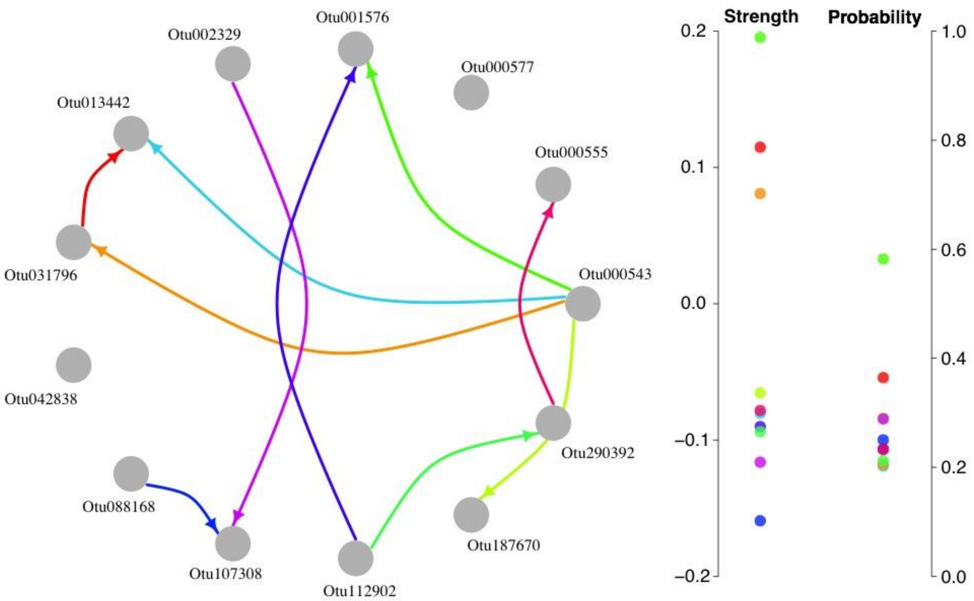

e. *Sarcotragus fasciculatus*

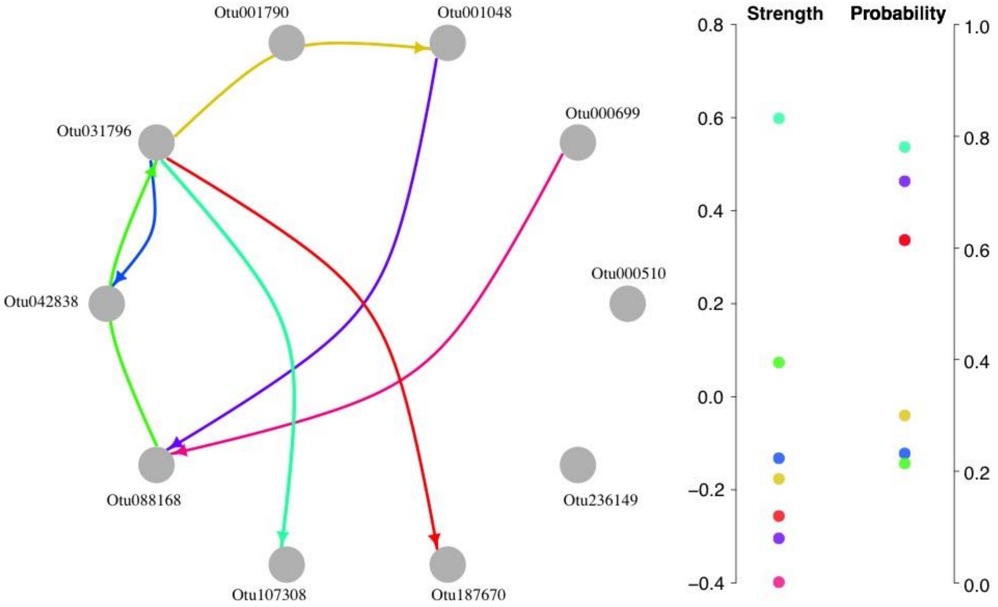

Supplementary Figure 7: The representative interspecific network for each core microbiome. The links in these networks corresponds to the most probable links shown in Supplementary Figure 4. Each link's colour is mapped to a certain strength and probability displayed on the scales to the right. Note that the scale for the interaction strength can range between negative and positive integers, while the scale for probability ranges from 0 to 1.

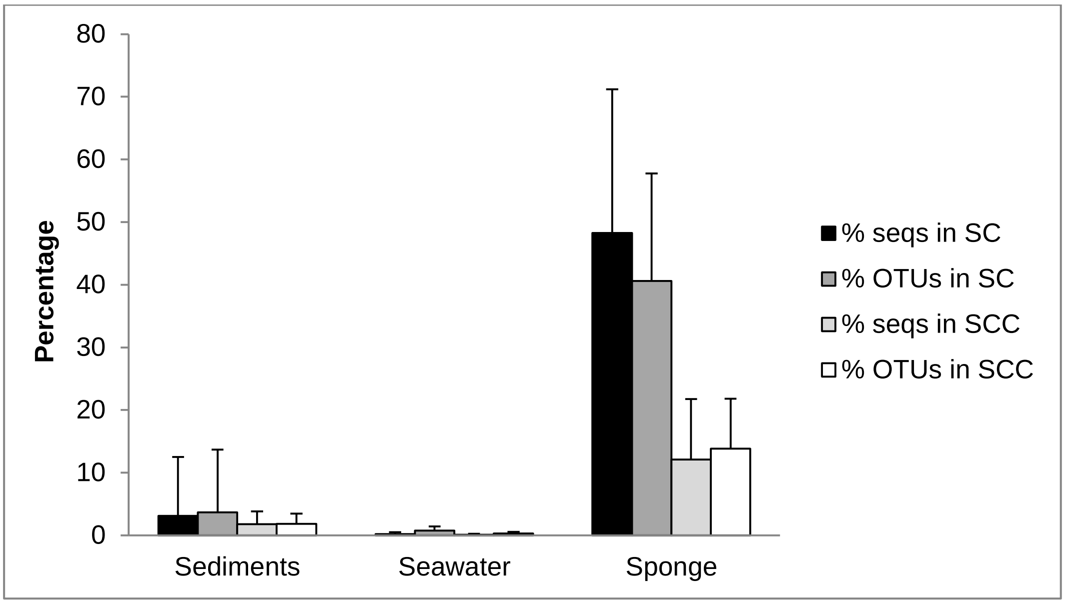

Supplementary Figure 8: Relative proportion of 16S rRNA gene sequences or OTUs that are assigned to sponge-specific clusters (SC) or sponge/coral-specific clusters (SCC) in sediment, seawater or sponge samples.

Supplementary Table 1: The percentage of total variation explained by inter-specific, density-dependent and environmental stochastic processes across the core microbiome of different sponge species.

Host species	Inter-specific interactions	Density dependence	Environmental Stochasticity
Carteriospongia foliascens	2.52	57.23	40.26
Cliona delitrix	2.55	57.95	39.50
Ircinia oros	5.39	72.54	22.07
Ircinia variabilis	7.09	55.83	37.08
Sarcotragus fasciculatus	7.76	63.76	28.48

Supplementary Table 2: Network properties for the core microbiomes of five sponge species.

Host species	Number of OTUs in core	Number of links	Connectance	Interaction strength
Carteriospongia foliascens	7	2.058 ± 1.275	4.900% ± 3.036%	0.019 ± 0.062
Cliona delitrix	8	3.246 ± 1.596	5.796% ± 2.849%	0.022 ± 0.050
Ircinia oros	20	17.986 ± 4.313	4.700% ± 1.100%	0.013 ± 0.043
Ircinia variabilis	13	9.800 ± 2.885	6.282% ± 1.849%	0.018 ± 0.030
Sarcotragus fasciculatus	10	6.726 ± 2.128	7.473% ± 2.364%	0.027 ± 0.083

Number of links, connectance and the interaction strength are calculated from the posterior distribution of the interaction coefficient alpha (Eq. 1 in Methods) and reported as the mean ± standard deviation.
